# 4‑*O*‑Allyl Derivative of Natural Licarin A Exhibits Selective *In Vitro* Activity against *Toxoplasma gondii* Tachyzoites

**DOI:** 10.1021/acsomega.6c05944

**Published:** 2026-10-01

**Authors:** Gabriel Candido Moura, Stephanie Ortega Alves, Ingrid de Oliveira Dias, João Paulo dos Santos Fernandes, Daniela da Costa Tristão, João Henrique Ghilardi Lago, Juliana Quero Reimão

**Affiliations:** † Laboratory of Preclinical Assays and Research of Alternative Sources of Innovative Therapy for Toxoplasmosis and Other Sicknesses (PARASITTOS), 146840 Faculdade de Medicina de Jundiaí, Jundiaí, São Paulo 13202-550, Brazil; ‡ Institute of Environmental, Chemical and Pharmaceutical Sciences, Universidade Federal de São Paulo, 09972-270 Diadema, São Paulo, Brazil; § Center of Natural and Human Sciences, Federal University of the ABC, 09210-580 Santo Andre, São Paulo, Brazil

## Abstract

*Toxoplasma gondii* is an obligate intracellular protozoan responsible for toxoplasmosis, a widespread parasitic disease that poses a serious health threat, especially to immunocompromised patients and pregnant women. Current therapeutic options remain unsatisfactory because they are associated with adverse effects and do not eliminate latent infections, emphasizing the need for new antitoxoplasmic agents. In the present study, the natural neolignan (−)-licarin A (**1**), isolated from the Brazilian species *Nectandra oppositifolia*, together with three semisynthetic derivatives, 4-*O*-methyl (**1a**), 4-*O*-acetyl (**1b**), and 4-*O*-allyl (**1c**), were investigated for their in vitro activity against *T. gondii* tachyzoites. Antiparasitic activity (EC_50_) was determined using a β-galactosidase-based assay, while cytotoxicity (CC_50_) toward Human Foreskin Fibroblasts (HFF) was assessed by the resazurin assay. Among the tested compounds, derivative **1c** exhibited the highest potency (EC_50_ = 3.49 μM) and lowest cytotoxicity (CC_50_ = 120.10 μM), resulting in the highest selectivity index (SI = 34.41). *In silico* ADMET analysis predicted favorable gastrointestinal absorption and blood–brain barrier permeability for all licarin derivatives. Mechanistic assays demonstrated that licarin A derivatives **1a**–**1c** did not compromise parasite plasma membrane integrity but significantly reduced mitochondrial membrane potential, supporting an intracellular mode of action. Further functional analyses revealed that compound **1c** inhibited intracellular parasite proliferation in a concentration-dependent manner and maintained significant antiparasitic activity after drug removal only at the highest concentration tested, without affecting parasite invasion or intracellular replication following pretreatment of extracellular tachyzoites. Overall, these findings identify the 4-*O*-allyl derivative of licarin A as the most selective compound of this series and provide additional evidence that semisynthetic modification of the licarin scaffold represents a promising strategy for discovering new therapeutic candidates against *T. gondii*.

## Graphical abstract

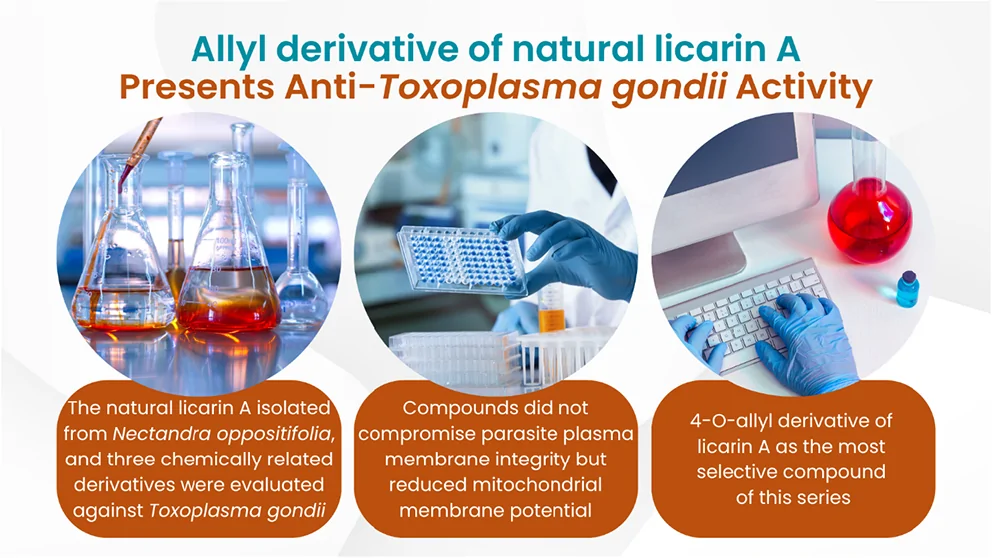

## Introduction

*Toxoplasma gondii* is an obligate intracellular protozoan parasite with worldwide distribution and is the etiological agent of toxoplasmosis, one of the most prevalent parasitic infections in humans, affecting approximately one-third of the global population.^1^ In healthy individuals, infection generally remains asymptomatic or manifests as a mild, self-limiting illness. However, severe clinical outcomes may occur in immunocompromised patients, particularly following reactivation of latent tissue cysts, which can result in toxoplasmic encephalitis and other life-threatening complications.^2^ In addition, congenital toxoplasmosis remains a significant clinical challenge, as transplacental transmission can result in miscarriage, severe fetal abnormalities, and long-term neurological or ocular sequelae.^3^

Despite its high prevalence and clinical relevance, the therapeutic options for toxoplasmosis remain limited and have changed little over the past decades. The standard therapeutic regimen combines pyrimethamine with sulfadiazine to inhibit folate biosynthesis in the parasite.^4^ However, this regimen is associated with important drawbacks, including significant toxicity, such as bone marrow suppression and hypersensitivity reactions, as well as poor tolerability in prolonged treatments.^5^ Importantly, these drugs are ineffective against the latent bradyzoite stage, precluding complete parasite clearance and allowing for disease reactivation. In this scenario, the identification of new compounds that are safer, more effective, and capable of targeting multiple parasite stages remains an urgent unmet need.^6^

Natural products continue to represent an invaluable source of structurally diverse molecules for drug discovery, including the identification of new antiparasitic agents. Several clinically important drugs, such as quinine and artemisinin, originated from plant-derived compounds, highlighting the potential of natural scaffolds as sources of therapeutic agents.^7^ In this context, the exploration of plant secondary metabolites represents a rational strategy to identify molecules capable of interfering with essential and often parasite-specific metabolic pathways.^8^ Among these natural compounds, lignans and neolignans have attracted increasing attention due to their broad spectrum of biological activities, including antimicrobial and antiparasitic effects.^9–14^ Within this class, the dihydrobenzofuran neolignan (−)-licarin A (**1**) has demonstrated promising activity against a variety of parasites, including *Leishmania* spp., *Trypanosoma cruzi*, and *Schistosoma mansoni*.^11,12,14^ Semisynthetic derivatives of licarin A have also shown improved antiparasitic activity and selectivity against protozoan parasites compared with the parent compound, highlighting the potential of structural modifications to optimize the biological properties of this scaffold.^12^ In particular, the 4-*O*-allyl derivative exhibited enhanced activity against *T. cruzi*, whereas other derivatives have demonstrated potent activity against *Leishmania infantum* by inducing alterations in calcium homeostasis and mitochondrial bioenergetics.^11,14^

More recently, a structurally related neolignan, licarin B, was reported to inhibit the proliferation of *T. gondii* tachyzoites by inducing mitochondrial dysfunction, autophagosome formation, and nuclear disorganization.^14,23^ Although these findings support the potential of licarin-based compounds as anti-*T. gondii* agents, the antiparasitic activity of semisynthetic derivatives of licarin A against this parasite has not yet been systematically investigated. Therefore, it remains unclear whether chemical modifications of the licarin A scaffold can improve its antitoxoplasmic activity and selectivity while preserving its favorable pharmacological properties.

Considering the urgent need for novel anti-*T. gondii* therapies and the promising bioactivity of neolignan derivatives, the present study aimed to evaluate the *in vitro* antiparasitic activity and cytotoxicity of the natural dihydrobenzofuran neolignan (−)-licarin A (**1**), isolated from *Nectandra oppositifolia*, together with three semisynthetic analogues, namely 4-*O*-methyl (**1a**), 4-*O*-acetyl (**1b**), and 4-*O*-allyl (**1c**), as shown in Figure 1. Following the initial screening, the derivative exhibiting the highest selectivity was further investigated through functional assays and mitochondrial membrane potential analysis to provide additional insights into its biological effects on *T. gondii*. By comparing the antiparasitic activity of these structurally related molecules, this study contributes to the identification of promising lead compounds for the development of new therapeutic strategies against toxoplasmosis.

**Figure 1:**
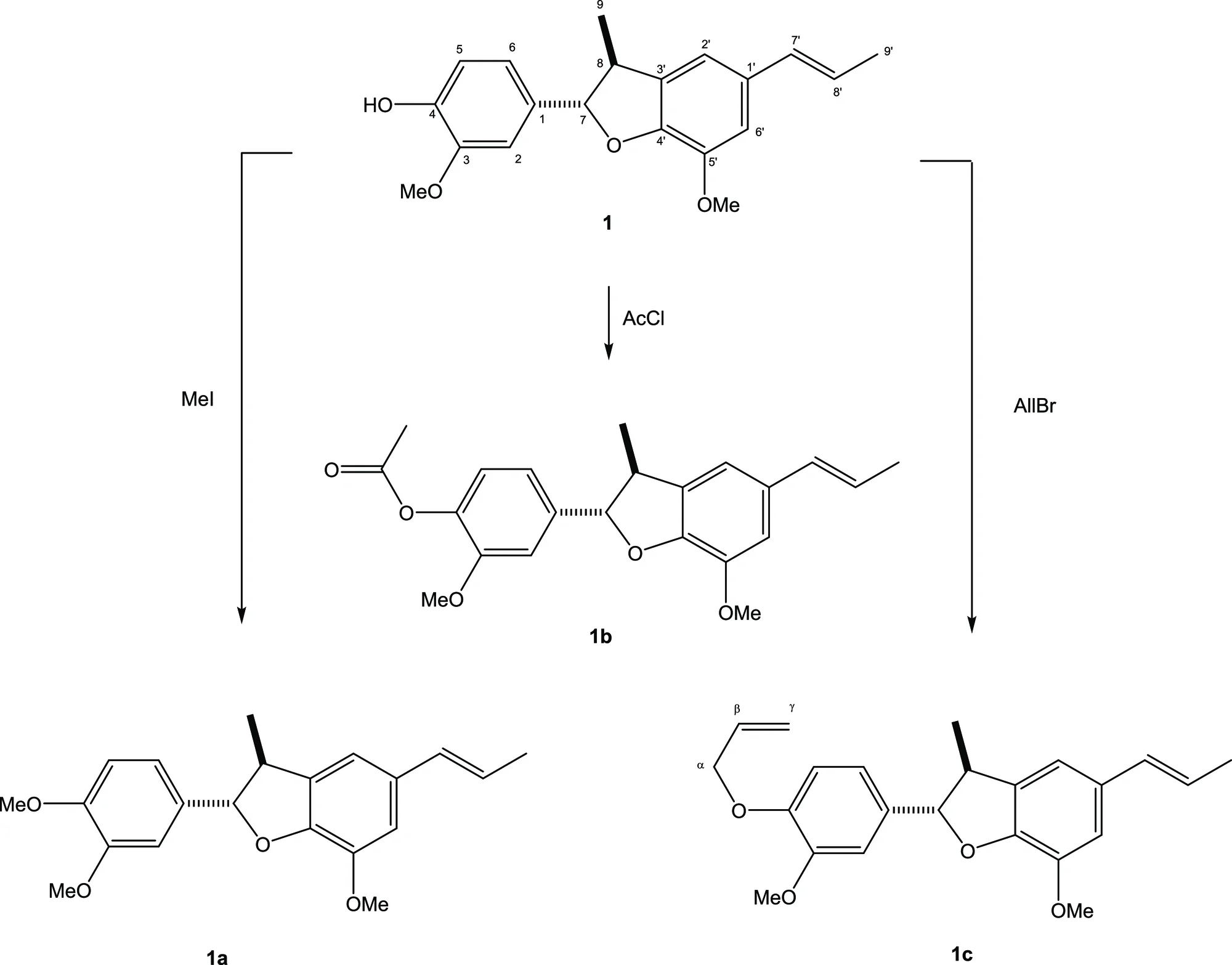
Structures of (−)-licarin A (**1**) and derivatives **1a**–**1c**.

## Results

The predicted pharmacokinetic and toxicity profiles of (−)-licarin A (**1**) and derivatives **1a**–**1c** are summarized in Table 1. All compounds are expected to display high gastrointestinal absorption together with the capacity to permeate the blood–brain barrier, suggesting favorable oral bioavailability and potential distribution to the central nervous system. Regarding toxicity, all licarin derivatives were predicted to present low mutagenic potential and no irritant effects. However, high probabilities of tumorigenic and reproductive effects were predicted for all derivatives. Compared with the reference drug pyrimethamine, the licarin derivatives displayed a similar GI absorption and BBB permeability profile, while exhibiting lower predicted mutagenic and reproductive toxicity.

**Table 1:**
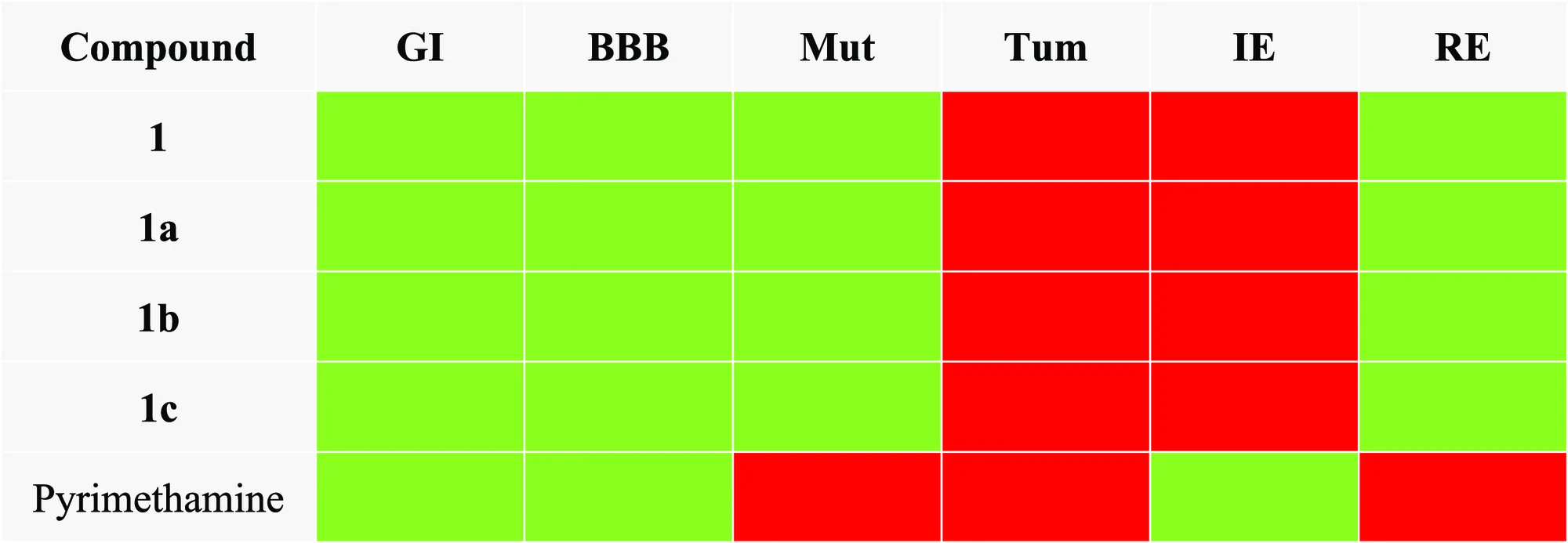
Predicted Pharmacokinetic and Toxicity Properties of (−)-Licarin A (**1**), Derivatives **1a**–**1c**, and Pyrimethamine^a^

a Green indicates favorable predicted properties, corresponding to high gastrointestinal (GI) absorption, blood–brain barrier (BBB) permeability, and the absence of predicted mutagenic, tumorigenic, irritant, or reproductive toxicity. Red indicates unfavorable predicted properties, corresponding to low GI absorption, lack of BBB permeability, or the presence of predicted toxicity risks.

The antiparasitic activity of (−)-licarin A (**1**) and derivatives **1a**–**1c** was determined using a β-galactosidase reporter assay. Cytotoxicity toward human foreskin fibroblasts (HFF) host cells was assessed by the resazurin viability assay, as summarized in Table 2.

**Table 2:** Half-Maximal Effective Concentration (EC_50_) against *T. gondii* Intracellular Tachyzoites and Half-Maximal Cytotoxic Concentration (CC_50_) of (−)-Licarin A (**1**) and Its Derivatives **1a**–**1c** and the Reference Drug Pyrimethamine

compound	EC_50_ (μM) ± SD against *T. gondii* tachyzoites	CC_50_ (μM) ± SD in human foreskin fibroblasts	selectivity index (CC_50_/EC_50_)
**1**	6.97 ± 2.36	41.96 ± 14.28	6.02
**1a**	5.46 ± 1.74	55.19 ± 12.46	10.11
**1b**	7.29 ± 1.88	52.30 ± 10.31	7.17
**1c**	3.49 ± 0.94	120.10 ± 24.23	34.41
Pyrimethamine	0.37 ± 0.03	>50	>135

All tested compounds exhibited antiparasitic activity, with EC_50_ values ranging from 3.49 to 7.29 μM. Although compound **1c** displayed the lowest EC_50_ value (3.49 ± 0.94 μM), the remaining derivatives also showed comparable micromolar activity, with EC_50_ values varying between 5.46 and 7.29 μM. Among the derivatives, the 4-*O*-allyl derivative (**1c**) was the most potent, followed by the 4-*O*-methyl derivative (**1a**), whereas the 4-*O*-acetyl derivative (**1b**) exhibited activity comparable to that of the naturally occurring compound **1**.

Cytotoxicity analysis revealed similar toxicity profiles for **1** and derivatives **1a** and **1b**, with CC_50_ values of 41.96 ± 14.28, 55.19 ± 12.46, and 52.30 ± 10.31 μM, respectively. In contrast, **1c** exhibited a higher CC_50_ value (120.10 ± 24.23 μM), suggesting that the presence of the allyl group not only enhances antiparasitic activity but also reduces cytotoxicity. Consequently, the superior selectivity of compound **1c** resulted primarily from its lower cytotoxicity combined with the lowest EC_50_ among the tested derivatives, yielding the highest selectivity index (SI = 34.41). Nevertheless, the reference drug pyrimethamine showed superior potency and selectivity, with an EC_50_ value of 0.37 ± 0.03 μM and a SI > 135.

To investigate whether compounds **1a**–**1c** affect parasite plasma membrane integrity, propidium iodide uptake assays were performed. As shown in Figure 2A, treatment with digitonin (positive control) resulted in a time-dependent increase in fluorescence intensity, consistent with progressive membrane permeabilization. In contrast, tachyzoites treated with (−)-licarin A and derivatives **1a**–**1c** displayed stable fluorescence levels over time, comparable to the DMSO-treated negative control, indicating preservation of membrane integrity. Quantitative analysis at 30 min (Figure 2B) confirmed these observations, revealing a significant increase in fluorescence exclusively in the positive-control group (*p* < 0.01), whereas none of the licarin-treated groups differed significantly from the negative control.

**Figure 2:**
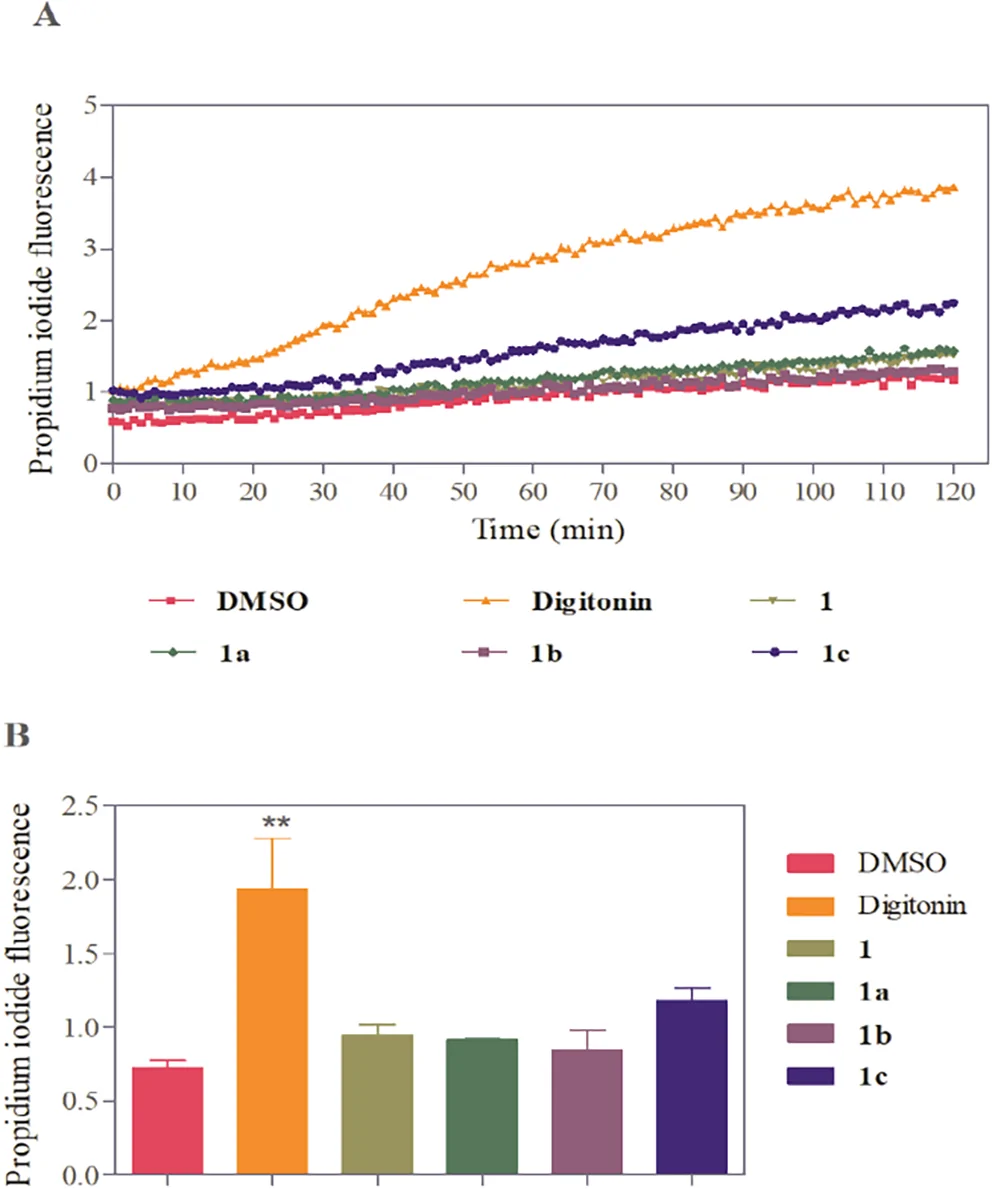
Effect of (−)-licarin A (**1**) and derivatives **1a**–**1c** on *T. gondii* plasma membrane integrity. (A) Time-course analysis of propidium iodide uptake in tachyzoites treated with licarin A (**1**) and derivatives **1a**–**1c** or digitonin at concentrations equivalent to their respective EC_50_ values. Fluorescence intensity was recorded every minute. (B) Quantification of fluorescence intensity after 30 min of treatment. Digitonin and DMSO were used as positive and negative controls for membrane permeabilization, respectively. Statistical significance was calculated relative to the DMSO-treated negative control. ** indicates *p* < 0.01.

Considering that plasma membrane integrity was preserved after compound treatment, mitochondrial membrane potential was evaluated to investigate whether licarin derivatives affect parasite mitochondrial function. As shown in Figure 3, treatment with the positive control antimycin A caused a marked reduction in MitoTracker Red fluorescence compared with the DMSO control (*p* < 0.001), confirming mitochondrial depolarization. Likewise, treatment with **1** and derivatives **1b** and **1c** at their respective EC_50_ values significantly decreased fluorescence intensity relative to the negative control (*p* < 0.05), indicating disruption of mitochondrial membrane potential. In contrast, no significant alteration was observed for the **1a** derivative. These results demonstrate that selected licarin derivatives affect mitochondrial function without causing detectable plasma membrane damage.

**Figure 3:**
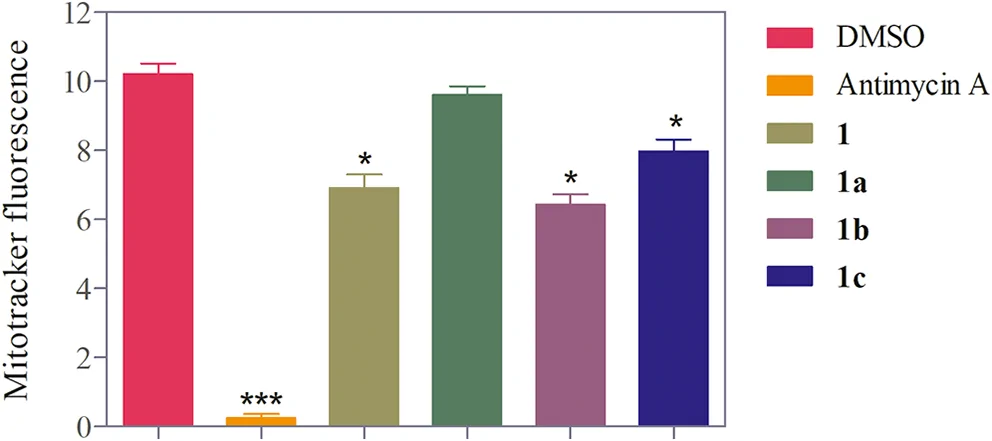
Effect of (−)-licarin A (**1**) and derivatives **1a**–**1c** on *T. gondii* mitochondrial membrane potential. Bar graph showing the fluorescence intensity of tachyzoites labeled with MitoTracker Red FM (100 nM) after treatment with **1** and derivatives **1a**–**1c** at their respective EC_50_ values, or antimycin A (10 μM). DMSO (0.01%) was used as the negative control. Statistical significance was determined relative to the DMSO-treated control (**p < 0.05; ***p < 0.001).

Given its superior selectivity, compound **1c** was further evaluated in functional assays assessing parasite proliferation, reversibility of drug effect, and host cell invasion. As shown in Figure 4A, compound **1c** significantly inhibited tachyzoite proliferation at concentrations of 30, 15, and 7.5 μM compared with the DMSO-treated control (*p* < 0.001). No differences were observed at concentrations of 3.75 μM or lower, despite the expected biological variability among independent experiments.

**Figure 4:**
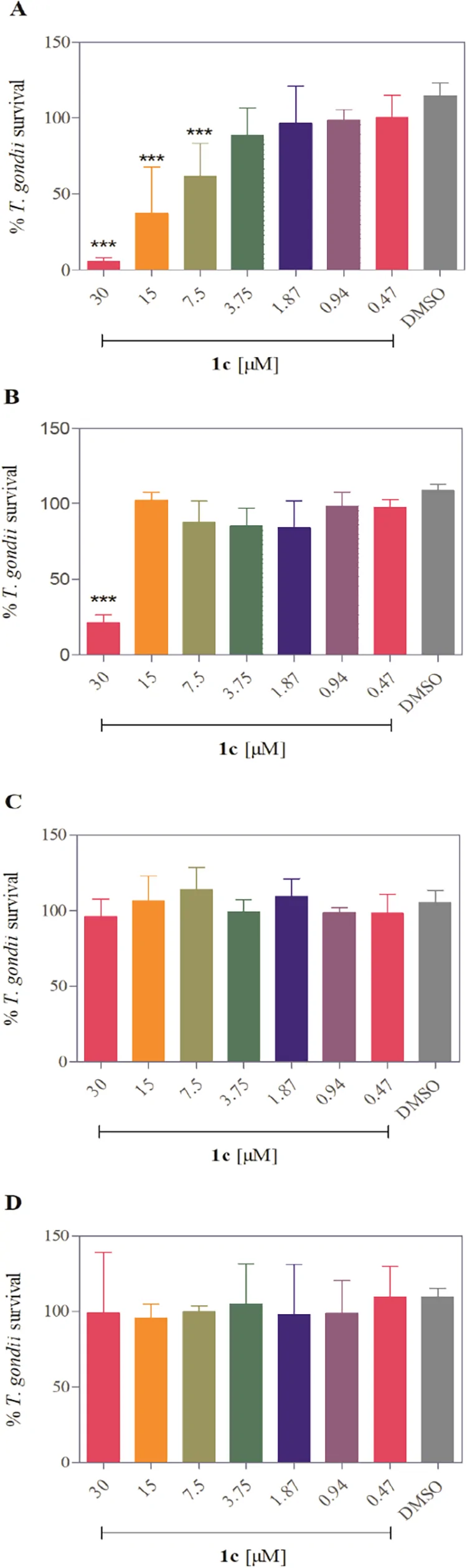
Functional characterization of the antiparasitic effects of the 4-*O*-allyl derivative (**1c**) against *T. gondii*. (A) Concentration-dependent inhibition of intracellular parasite proliferation. (B) Evaluation of parasite recovery following 24 h exposure to the compound and subsequent incubation for 48 h in compound-free medium. (C) Host-cell invasion assay performed using extracellular tachyzoites pre-exposed to **1c** for 1 h before infection. (D) Intracellular replication of parasites pretreated prior to host-cell infection. Statistical comparisons were performed using the DMSO-treated group as reference (***p < 0.001).

In the reversibility assay (Figure 4B), only the highest concentration tested (30 μM) resulted in a significant reduction in parasite recovery following drug removal (*p* < 0.001), indicating that exposure to high concentrations of compound **1c** produced sustained antiparasitic effects after drug withdrawal. Lower concentrations did not differ significantly from the DMSO-treated control.

Pretreatment of extracellular tachyzoites with compound **1c** did not significantly influence their ability to invade host cells (Figure 4C). Likewise, intracellular replication assessed 72 h after infection (Figure 4D) remained unaffected, indicating that transient exposure of extracellular parasites to the compound is insufficient to impair subsequent invasion or intracellular multiplication.

## Discussion

Neolignans comprise a structurally diverse group of plant-derived metabolites that have attracted considerable interest because of their broad spectrum of biological activities, including activity against both helminth and protozoan parasites.^15–19^ In this context, (−)-licarin A (**1**) and several derivatives have been previously reported as promising scaffolds against parasites such as *S. mansoni*,^14,20^ *T. cruzi*,^12,14,21^ *Leishmania* spp.,^11,22^ and the structurally related neolignan licarin B has demonstrated activity against *T. gondii*,^23^ supporting the exploration of this chemical class as a valuable source of lead compounds for antiparasitic drug discovery. The present study expands the findings by providing the first systematic evaluation of semisynthetic licarin A derivatives against *T. gondii*, demonstrating that all tested compounds display antiparasitic activity, while the 4-*O*-allyl derivative (**1c**) exhibits the most favorable balance between efficacy and host cell safety.

The observed EC_50_ values in the low micromolar range are consistent with those reported for licarin A derivatives **1a**–**1c** against other protozoan parasites. For instance, these derivatives exhibited EC_50_ values between 9 and 13 μM against intracellular amastigotes of *L. infantum*,^11^ whereas the 4-*O*-allyl derivative previously showed improved activity against bloodstream forms of *T. cruzi*.^12^ Although compound **1c** exhibited the lowest EC_50_ in the present study, the differences in antiparasitic potency among the evaluated derivatives were relatively modest. In contrast, the substantially lower cytotoxicity of compound **1c** resulted in a markedly higher selectivity index, which ultimately supported its selection for subsequent mechanistic investigations. This finding reinforces previous structure–activity relationship studies indicating that semisynthetic modifications of the licarin scaffold can improve its biological profile without substantially altering its antiparasitic potency.^12^

The *in silico* ADMET analysis further supports the potential of this chemical class for antiparasitic drug discovery. All licarin derivatives were predicted to possess high gastrointestinal absorption together with the ability to penetrate the blood–brain barrier, two desirable properties for compounds intended to treat cerebral toxoplasmosis. Moreover, the predicted absence of mutagenicity and irritant effects represents a favorable safety feature, although the predicted tumorigenic and reproductive risks highlight the need for comprehensive toxicological evaluation in future preclinical studies. These computational predictions should be interpreted cautiously, as they require experimental validation.

In addition to potency, selectivity toward the parasite over host cells is a critical parameter for drug development. 4-*O*-allyl licarin A (**1c**) exhibited a markedly higher selectivity index compared to the parent compound (−)-licarin A (**1**), 4-*O*-methyl (**1a**) and 4-*O*-acetyl (**1b**) derivatives. Although the reference drug pyrimethamine remains significantly more potent and selective, the selectivity index obtained for compound **1c** is within a range considered promising for hit compounds in early stage drug discovery.

Mechanistically, the absence of plasma membrane permeabilization in tachyzoites treated with licarin derivatives indicates that their antiparasitic effect is not mediated by direct membrane disruption. This finding is consistent with previous reports in *Leishmania*, in which structurally related licarin derivatives also failed to induce plasma membrane damage.^11^ To further investigate their intracellular effects, mitochondrial membrane potential was evaluated in the present study. (−)-Licarin A (**1**), compound **1b**, and particularly compound **1c** significantly reduced MitoTracker fluorescence, indicating disruption of mitochondrial membrane potential. Together with the preserved plasma membrane integrity, these findings suggest that mitochondrial dysfunction represents an early cellular event associated with the antiparasitic activity of these compounds.

Our results are also consistent with previous mechanistic studies in other protozoan parasites. In *T. cruzi*, licarin-derived compounds induced mitochondrial depolarization, oxidative stress, and alterations in ATP homeostasis,^12^ whereas in *L. infantum* they promoted mitochondrial dysfunction associated with calcium imbalance and bioenergetic impairment. Likewise, licarin B was previously shown to induce mitochondrial damage, autophagosome formation, and nuclear disorganization in *T. gondii*.^23^ Although the present study did not investigate ROS production, ATP depletion, or ultrastructural alterations, the observed mitochondrial depolarization strengthens the evidence that mitochondria constitute an important intracellular target of licarin-based compounds across different protozoan parasites.

The lack of effect on host cell invasion observed in the present study further indicates that compound **1c** does not impair early host–parasite interactions. Likewise, pretreatment of extracellular parasites did not affect their subsequent intracellular proliferation, suggesting that brief exposure to the compound before infection is insufficient to induce irreversible damage. In contrast, continuous treatment of infected cells significantly reduced parasite proliferation, indicating that compound **1c** exerts its antiparasitic activity predominantly after parasite internalization. Furthermore, the reversibility assay demonstrated that only the highest concentration tested maintained significant inhibition following compound removal, indicating that the sustained antiparasitic effect depends on drug concentration and exposure time rather than representing a fully irreversible mechanism of action.

Taken together, these findings indicate that semisynthetic modification of the licarin scaffold preserves antiparasitic activity while improving selectivity and provides additional evidence supporting mitochondrial dysfunction as a relevant cellular target. Nevertheless, complementary studies evaluating reactive oxygen species production, ATP levels, ultrastructural changes, and molecular targets will be required to obtain a comprehensive understanding of the mechanism of action of these compounds.

## Conclusions

Collectively, the present results demonstrate that natural (−)-licarin A (**1**) and its semisynthetic derivatives **1a**–**1c** exhibit consistent *in vitro* activity against *T. gondii* tachyzoites. Although all compounds displayed similar micromolar antiparasitic activity, the 4-*O-*allyl derivative (**1c**) combined the lowest cytotoxicity with the highest selectivity index, making it the most promising candidate for further investigation.

Mechanistic assays showed that the antiparasitic activity was not associated with plasma membrane disruption but was accompanied by mitochondrial membrane depolarization, supporting mitochondrial dysfunction as a potential mechanism involved in parasite killing. In addition, the predicted pharmacokinetic profile suggests favorable gastrointestinal absorption and blood–brain barrier permeability, characteristics that may be advantageous for future development of antitoxoplasmosis agents, although these predictions require experimental confirmation.

Future studies should focus on elucidating the precise molecular targets in *T. gondii*, validating the mitochondrial effects through complementary biochemical approaches, and evaluating pharmacokinetic properties, safety, and *in vivo* efficacy to advance these compounds toward preclinical development.

## Methods

### General Experimental Procedures

Column chromatographic separations were carried out on silica gel 60, and reaction progress was monitored by thin-layer chromatography (TLC) on silica gel plates containing a fluorescent indicator, with spots visualized under UV light at 254 nm. Stock solutions (10 mM) were prepared in dimethyl sulfoxide (DMSO) and diluted in culture medium to the desired concentrations immediately prior to use. All additional reagents were purchased from Sigma-Aldrich unless otherwise stated. NMR spectra were recorded on a Bruker Avance 300 spectrometer operating at 300 and 75 MHz for the ^1^H and ^13^C nuclei, respectively, using CDCl_3_ as the solvent and TMS as the internal standard. Chemical shifts (δ) are reported in ppm, and coupling constants (*J*) in Hz. HRESIMS data were acquired using a Bruker Daltonics MicroTOF QII mass spectrometer. Optical rotations were measured with a JASCO P-2000 polarimeter equipped with a sodium filter (λ = 589 nm).

### Plant Material

Fresh leaves of *N. oppositifolia* were collected in June 2023 in the municipality of Artur Nogueira, São Paulo State, Brazil, from the same specimen previously investigated.^12^ The voucher specimen was compared with the reference material deposited at the Herbarium of the Institute of Biosciences, University of São Paulo, São Paulo, Brazil, under accession number SPF225339.

### Isolation of (−)-Licarin A from *N. oppositifolia*

After drying and pulverization, the leaves of *N. oppositifolia* (600 g) were exhaustively extracted with hexanes at room temperature. The combined extracts were concentrated under reduced pressure to yield 65 g of crude hexane extract. The extract was subjected to silica gel column chromatography, using hexanes with increasing proportions of EtOAc as the mobile phase, affording six fractions (I–VI). Fraction IV (6.2 g) was further purified by silica gel column chromatography, eluted with hexanes/EtOAc (7:3), to afford 2.7 g of (−)-licarin A.

#### (−)-Licarin A (**1**)

[α]_D_24 −63 (*c* 0.11, MeOH). ^1^H NMR (CDCl_3_), δ/ppm: 6.97 (d, *J* = 1.4 Hz, H-2), 6.92 (dd, *J* = 7.8 and 1.4 Hz, H-6), 6.91 (d, *J* = 7.8 Hz, H-5), 6.79 (br s, H-2′), 6.77 (br s, H-6′), 6.36 (dd, *J* = 15.7 and 1.6 Hz, H-7′), 6.11 (dq, *J* = 15.7 and 6.6 Hz, H-8′), 5.63 (s, OH), 5.11 (d, *J* = 9.4 Hz, H-7), 3.90 (s, OCH_3_), 3.89 (s, OCH_3_), 3.45 (dq, *J* = 9.4 and 6.8 Hz, H-8), 1.87 (dd, *J* = 6.6 and 1.6 Hz, H-9′), 1.38 (d, *J* = 6.8 Hz, H-9). ^13^C NMR (CDCl_3_), δ/ppm: 146.7 (C-3), 146.6 (C-4′), 145.8 (C-4), 144.2 (C-5′), 132.2 (C-1′), 132.1 (C-1), 130.9 (C-7′), 123.5 (C-8′), 120.0 (C-6), 114.1 (C-5), 113.3 (C-2′), 113.2 (C-3′), 109.3 (C-6′), 108.9 (C-2), 93.8 (C-7), 56.1 (OCH_3_), 56.0 (OCH_3_), 45.6 (C-8), 18.4 (C-9′), 17.6 (C-9). HRESIMS *m*/*z* 325.8384 [M – H]^−^ (calcd for C_20_H_21_O_4_ 325.1439).

### Procedure for the Preparation of Compound **1a**

Compound **1** (250 mg) was treated with K_2_CO_3_ (2 equiv) and CH_3_I (2 equiv) in acetone (10 mL) under reflux for 7 h. The solvent was then removed under reduced pressure, and the residue was extracted with CH_2_Cl_2_ and washed with H_2_O (2 × 25 mL). The organic phase was dried over anhydrous Na_2_SO_4_ and concentrated under reduced pressure. The resulting residue was purified by silica gel column chromatography using *n*-hexane/EtOAc (9:1) as the eluent to afford **1a** as a white amorphous solid in 98% yield.

#### 4-*O*-Methyl licarin A (**1a**)

^1^H NMR (CDCl_3_) δ/ppm: 6.98 (d, *J* = 1.7 Hz, H-2), 6.88 (dd, *J* = 7.8 and 1.7 Hz, H-6), 6.95 (d, *J* = 7.8 Hz, H-5), 6.86 (d, *J* = 1.8 Hz, H-6′), 6.80 (d, *J* = 1.8 Hz, H-2′), 6.36 (dd, *J* = 15.7 and 1.3 Hz, H-7′), 6.10 (dq, *J* = 15.7 and 6.5 Hz, H-8′), 5.11 (d, *J* = 9.4 Hz, H-7), 3.91 (s, OCH_3_), 3.90 (s, OCH_3_), 3.88 (s, OCH_3_), 3.45 (dd, *J* = 9.4 and 6.8 Hz, H-8), 1.86 (dd, *J* = 6.8 and 1.3 Hz, H-9′), 1.38 (d, *J* = 6.8 Hz, H-9). ^13^C NMR (CDCl_3_) δ/ppm: 149.1 (C-4), 149.0 (C-5′), 146.6 (C-6′), 144.2 (C-3), 133.3 (C-4′), 132.6 (C-6), 132.2 (C-6′), 130.9 (C-7′), 123.5 (C-8′), 119.2 (C-1), 113.3 (C-2), 110.8 (C-3′), 109.5 (C-1′), 108.9 (C-5), 93.6 (C-7), 55.9 (OCH_3_), 55.8 (OCH_3_), 55.9 (OCH_3_), 45.5 (C-8), 18.4 (C-9′), 17.6 (C-9). HRESIMS *m*/*z* 341.1772 [M + H]^+^ (calcd. for C_21_H_25_O_4_ 341.1753).

### Procedure for the Preparation of Compound **1b**

Compound **1** (350 mg) was treated with Et_3_N (1 equiv) and acetyl chloride (2 equiv) in CH_2_Cl_2_ (5 mL) and stirred at 0 °C for 4 h. The reaction mixture was then treated with 20 mL of 10% aqueous NaHCO_3_, and the organic phase was separated and washed with H_2_O (2 × 25 mL). The organic layer was dried over anhydrous Na_2_SO_4_ and concentrated under reduced pressure. The residue was purified by silica gel column chromatography using *n*-hexane/EtOAc (9:1) as the eluent to afford **1b** as a white amorphous solid in 27% yield.

#### 4-*O*-Acetyl Licarin A (**1b**)

^1^H NMR (CDCl_3_) δ/ppm: 7.00 (br s, H-6′), 6.98 (br s, H-2′), 6.88 (br s, H-2), 6.80 (br s, H-6), 6.78 (br s, H-5), 6.36 (dd, *J* = 15.7 and 1.5 Hz, H-7′), 6.10 (dq, *J* = 15.7 and 6.6 Hz, H-8′), 5.11 (d, *J* = 9.2 Hz, H-7), 3.46 (dd, *J* = 9.2 and 6.8 Hz, H-8), 1.87 (dd, *J* = 6.6 and 1.5 Hz, H-9′), 1.41 (d, *J* = 6.8 Hz, H-9), 3.90 (s, OCH_3_), 3.88 (s, OCH_3_), 2.31 (s, CH_3_). ^13^C NMR (CDCl_3_) δ/ppm: 169.0 (C = O), 151.2 (C-5′), 146.5 (C-4), 144.1 (C-6′), 139.6 (C-3), 139.3 (C-4′), 133.0 (C-6), 132.4 (C-6′), 130.9 (C-7′), 123.6 (C-8′), 122.7 (C-1), 118.7 (C-2), 113.4 (C-3′), 110.3 (C-1′), 109.3 (C-5), 93.1 (C-7), 55.9 (OCH_3_), 45.8 (C-8), 17.9 (C-9), 20.7 (CH_3_), 18.4 (C-9′). HRESIMS *m*/*z* 369.1608 [M + H]^+^ (calcd. for C_22_H_25_O_5_ 369.1702).

### Procedure for the Preparation of Compound **1c**

Compound **1** (600 mg) was treated with K_2_CO_3_ (2 equiv) and allyl bromide (2 equiv) in acetone (15 mL) under reflux for 19 h. The solvent was then removed under reduced pressure, and the residue was extracted with CH_2_Cl_2_ and washed with H_2_O (2 × 25 mL). The organic phase was dried over anhydrous Na_2_SO_4_ and concentrated under reduced pressure. The resulting residue was purified by silica gel column chromatography using *n*-hexane/EtOAc (9:1) as the eluent to afford **1c** as a white amorphous solid in 73% yield.

#### 4-*O*-Allyl Licarin A (**1c**)

^1^H NMR (CDCl_3_) δ/ppm: 6.99 (d, *J* = 8.2 Hz, H-5), 6.93 (d, *J* = 1.9 Hz, H-2), 6.92 (dd, *J* = 8.2 and 1.9 Hz, H-6), 6.84 (d, *J* = 1.9 Hz, H-2′), 6.78 (d, *J* = 1.9 Hz, H-6′), 6.36 (dd, *J* = 15.7 and 1.5 Hz, H-7′), 6.09 (qdd, *J* = 10.6, 7.2, and 4.8 Hz, H-β), 6.04 – 6.16 (m, H-8′), 5.39 (dd, *J* = 17.3 and 1.5 Hz; H-γ), 5.11 (d, *J* = 9.4 Hz, H-7), 4.61 (dt, *J* = 5.4 and 1.4 Hz, H-α), 3.86 (s, OCH_3_), 3.89 (s, OCH_3_), 3.46 (dq, *J* = 9.4 and 6.8 Hz; H-8), 1.86 (dd, *J* = 6.6 and 1.5 Hz, H-9′), 1.38 (d, *J* = 6.8 Hz, H-9). ^13^C NMR (CDCl_3_) δ/ppm: 149.6 (C-5′), 148.1 (C-4′), 146.6 (C-6′), 144.1 (C-3), 133.3 (C-4), 133.2 (C-2), 133.0 (C-6), 132.3 (C-2′), 130.9 (C-7′), 123.5 (C-8′), 119.1 (C-1), 118.0 (C-γ), 113.3 (C-β), 113.1 (C-3′), 109.9 (C-1′), 109.2 (C-5), 93.6 (C-7), 69.9 (C-α), 55.9 (OCH_3_), 45.6 (C-8), 18.4 (C-9′), 17.7 (C-9). HRESIMS *m*/*z* 367.1920 [M + H]^+^ (calcd. for C_23_H_26_O_4_ 367.1909).

### Parasite and Host Cell Maintenance

The RH-2F1 strain of *T. gondii*, which constitutively expresses β-galactosidase and was originally generated by Seeber and Boothroyd,^24^ was propagated by continuous passage in human foreskin fibroblast (HFF) monolayers. HFF cells (ATCC SCRC-1041) were maintained in Dulbecco’s modified Eagle medium (DMEM) supplemented with 10% fetal bovine serum (FBS), 2 mM l-glutamine, and 40 μg/mL gentamicin (D10 medium). Cell cultures were incubated at 37 °C in a humidified incubator with 5% CO_2_, following previously established procedures.^25^ For parasite infection experiments, confluent HFF monolayers were switched to DMEM containing 2% FBS (D2 medium).

### Determination of Half-Maximal Effective Concentration (EC_50_)

Antiparasitic activity was quantified by monitoring β-galactosidase activity as an indirect measure of intracellular parasite growth, according to a previously reported protocol.^26^ Immediately after inoculation, infected HFF monolayers were exposed to compound **1** or derivatives **1a**–**1c** at concentrations ranging from 30 to 0.47 μM. Cultures were maintained for 72 h without removing extracellular parasites. At the end of the incubation period, cells were lysed for 15 min at room temperature with 100 μL of lysis buffer (100 mM HEPES, pH 7.5, 1 mM MgSO_4_, 0.1% Triton X-100, and 5 mM DTT). Enzymatic activity was then determined by adding 160 μL of assay buffer (100 mM sodium phosphate, pH 7.3, 102 mM β-mercaptoethanol, and 9 mM MgCl_2_) together with 40 μL of chlorophenol red-β-d-galactopyranoside (CPRG, 6.25 mM). Following a 30 min incubation at 37 °C, absorbance was recorded at 570 nm using a Varioskan LUX microplate reader (Thermo Scientific). Untreated infected cultures, vehicle-treated cultures (1% DMSO), pyrimethamine-treated cultures, and blank wells served as viability, vehicle, reference drug, and background controls, respectively. All assays were performed in duplicate in three independent experiments. Concentration–response curves were fitted by nonlinear regression using a four-parameter logistic model, and EC_50_ values were calculated with SkanIt software (Thermo Fisher Scientific).

### Determination of Half-Maximal Cytotoxic Concentration (CC_50_)

Cytotoxicity was assessed in uninfected HFF monolayers using the resazurin reduction assay as previously described.^27^ After complete monolayer formation, cells were incubated with compounds at the same concentration range employed in the antiparasitic assay (30–0.47 μM) for 72 h. Resazurin was subsequently added to a final concentration of 100 μM, and the plates were incubated for an additional 4 h at 37 °C. Fluorescence was measured at excitation and emission wavelengths of 565 and 590 nm, respectively. Untreated cells, vehicle-treated cells (1% DMSO), pyrimethamine-treated cells, and blank wells were included as controls. Experiments were carried out in duplicate and independently repeated three times. CC_50_ values were obtained by fitting the data to a four-parameter nonlinear regression model using SkanIt software.

### Plasma Membrane Integrity Assay

The integrity of the parasite plasma membrane following treatment with **1** and derivatives **1a**–**1c** was examined using propidium iodide uptake, according to a previously reported method.^28^ Freshly released tachyzoites (3 × 10^6^ parasites) were suspended in PBS and incubated for 30 min at 37 °C under 5% CO_2_ in the presence of each compound at its corresponding EC_50_ concentration. Digitonin (3 μM) was included as the positive control for membrane permeabilization, whereas parasites exposed to 0.01% DMSO served as the negative control. Fluorescence was monitored with a Varioskan LUX microplate reader (Thermo Fisher Scientific) using excitation and emission wavelengths of 504 and 524 nm, respectively.

### Mitochondrial Membrane Potential Assay

Changes in mitochondrial membrane potential were investigated using the fluorescent probe MitoTracker Red FM. Freshly egressed tachyzoites (2 × 10^6^ parasites per well) were incubated with compounds **1** and **1a**–**1c** at concentrations equivalent to their respective EC_50_ values for 30 min at 37 °C in a humidified atmosphere containing 5% CO_2_. Parasites treated with 0.01% DMSO or antimycin A (10 μM) were used as negative and positive controls, respectively. After treatment, parasites were washed with PBS and stained with 100 nM MitoTracker Red FM for an additional 30 min under the same incubation conditions. Fluorescence intensity was then determined using a Varioskan LUX multimode microplate reader (Thermo Fisher Scientific) with excitation and emission wavelengths set at 603 and 651 nm. Each experiment included eight technical replicates and was independently repeated three times.

### *In Vitro* Assays with 4-*O*-Allyl Licarin A (**1c**)

#### Parasite Proliferation Assay

HFF cells (1 × 10^4^ cells/well) were seeded in D10 medium and allowed to adhere overnight. Monolayers were subsequently infected with *T. gondii* tachyzoites at an MOI of 1:1 and maintained for 3 h to permit parasite invasion. After removal of extracellular parasites by PBS washing, infected cells were exposed to compound **1c** (30–0.47 μM) for 72 h. Parasite growth was quantified by measuring β-galactosidase activity using the procedure described above.^29^

#### Reversibility Assay

To determine whether the inhibitory activity of compound **1c** persisted after compound withdrawal, infected HFF monolayers were exposed to the compound for 24 h. The treatment medium was then replaced with fresh compound-free medium, and cultures were maintained for an additional 48 h. Parasite viability at the end of the experiment was quantified by the β-galactosidase assay.^29^

#### Invasion and Intracellular Proliferation Assays Using Pretreated Tachyzoites

Extracellular tachyzoites were incubated with compound **1c** (30–0.47 μM) for 1 h at 37 °C, washed thoroughly with PBS after centrifugation (1900*g*, 10 min), and immediately used to infect HFF monolayers at an MOI of 1:1. After 3 h, nonadherent parasites were removed by washing. Parasite invasion was quantified immediately after this step by β-galactosidase activity. To evaluate intracellular replication, parallel cultures were maintained for an additional 72 h before parasite growth was quantified using the same enzymatic assay.^29^

### *In Silico* ADMET Prediction

The pharmacokinetic and toxicity properties of **1** and derivatives **1a**–**1c** were predicted using freely available computational platforms. Gastrointestinal absorption (GI), blood–brain barrier permeability (BBB), lipophilicity, and drug-likeness descriptors were predicted using SwissADME (http://www.swissadme.ch). Toxicological end points, including mutagenicity, tumorigenicity, irritant effects, and reproductive toxicity, were estimated using the OSIRIS Property Explorer. These analyses were used to compare the predicted pharmacokinetic behavior and safety profiles of the evaluated compounds.

### Statistical Analysis

Experimental data are presented as mean ± SD obtained from at least three independent experiments. Statistical comparisons among groups were carried out by one-way ANOVA followed by Tukey’s multiple-comparison test, adopting *p* < 0.05 as the threshold for statistical significance. Dose–response curves and EC_50_/CC_50_ values were generated by fitting a four-parameter logistic nonlinear regression model using SkanIt software (Thermo Fisher Scientific).

## Supplementary Material



## Notes

HPLC analysis, HRESIMS and NMR spectra for compounds **1, 1a–1c**; HPLC analysis of compound **1** (Figure S1); HRESIMS spectrum (negative mode) of compound **1** (Figure S2); ^13^C NMR spectrum of compound **1** (δ/ppm, 75 MHz, CDCl_3_) (Figure S3); ^1^H NMR spectrum of compound **1** (δ/ppm, 300 MHz, CDCl_3_) (Figure S4); HPLC analysis of compound **1a** (Figure S5); HRESIMS spectrum (positive mode) of compound **1a** (Figure S6); ^13^C NMR spectrum of compound **1a** (δ/ppm, 75 MHz, CDCl_3_) (Figure S7); ^1^H NMR spectrum of compound **1a** (δ/ppm, 300 MHz, CDCl_3_) (Figure S8); HPLC analysis of compound **1b** (Figure S9); HRESIMS spectrum (positive mode) of compound **1b** (Figure S10); ^13^C NMR spectrum of compound **1b** (δ/ppm, 75 MHz, CDCl_3_) (Figure S11); ^1^H NMR spectrum of compound **1b** (δ/ppm, 300 MHz, CDCl_3_) (Figure S12); HPLC analysis of compound **1c** (Figure S13); HRESIMS spectrum (positive mode) of compound **1c** (Figure S14); ^13^C NMR spectrum of compound **1c** (δ/ppm, 75 MHz, CDCl_3_) (Figure S15); ^1^H NMR spectrum of compound **1c** (δ/ppm, 300 MHz, CDCl_3_) (Figure S16) (PDF)
