# Supplementary material for: 4‑O‑Allyl Derivative of Natural Licarin A Exhibits Selective In Vitro Activity against Toxoplasma gondii Tachyzoites
Source: ACS Omega. 2026 Oct 1;11(40):61199–207. doi: 10.1021/acsomega.6c05944 (PMC13670340; doi:10.1021/acsomega.6c05944)
Supplement: Supplementary file 1 [file ao6c05944_si_001.pdf]

4-*O*-Allyl derivative of natural licarin A exhibits selective *in vitro* activity against *Toxoplasma gondii* tachyzoites

Gabriel Candido Moura[1], Stephanie Ortega Alves[1], Ingrid de Oliveira Dias[1], João Paulo dos Santos Fernandes[2], Daniela da Costa Tristão[3], João Henrique Ghilardi Lago[3*], Juliana Quero Reimão[1*]

[1]Laboratory of Preclinical Assays and Research of Alternative Sources of Innovative Therapy for Toxoplasmosis and Other Sicknesses (PARASITTOS), Faculdade de Medicina de Jundiaí, 13202-550 Jundiaí, São Paulo, Brazil.

[2]Institute of Environmental, Chemical and Pharmaceutical Sciences, Universidade Federal de São Paulo, 09972-270 Diadema, São Paulo, Brazil.

[3]Center of Natural and Human Sciences, Federal University of ABC, 09210-580 Santo Andre, São Paulo, Brazil.

SUPPORTING INFORMATION

*Corresponding authors

João Henrique Ghilardi Lago (joao.lago@ufabc.edu.br)

Juliana Quero Reimão (julianareimao@g.fmj.br)

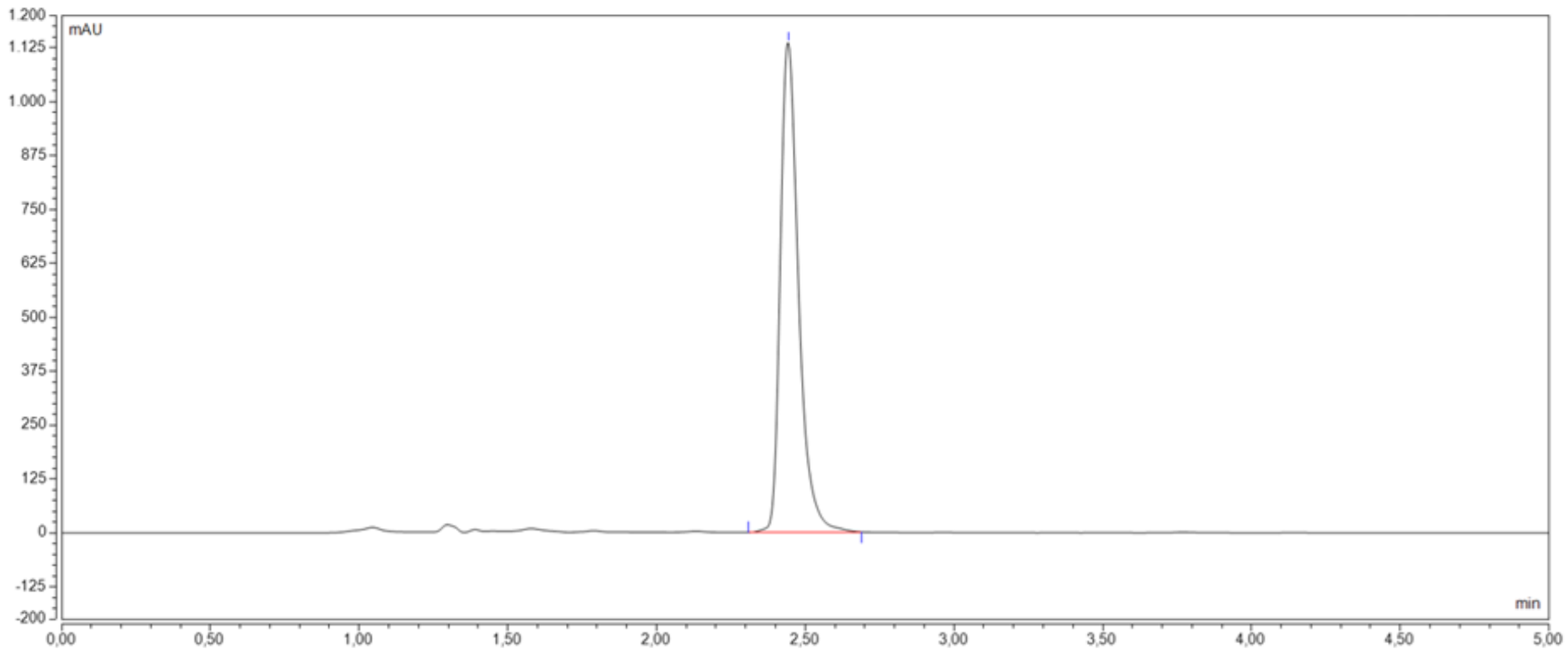

Figure S1. HPLC analysis of compound **1**.

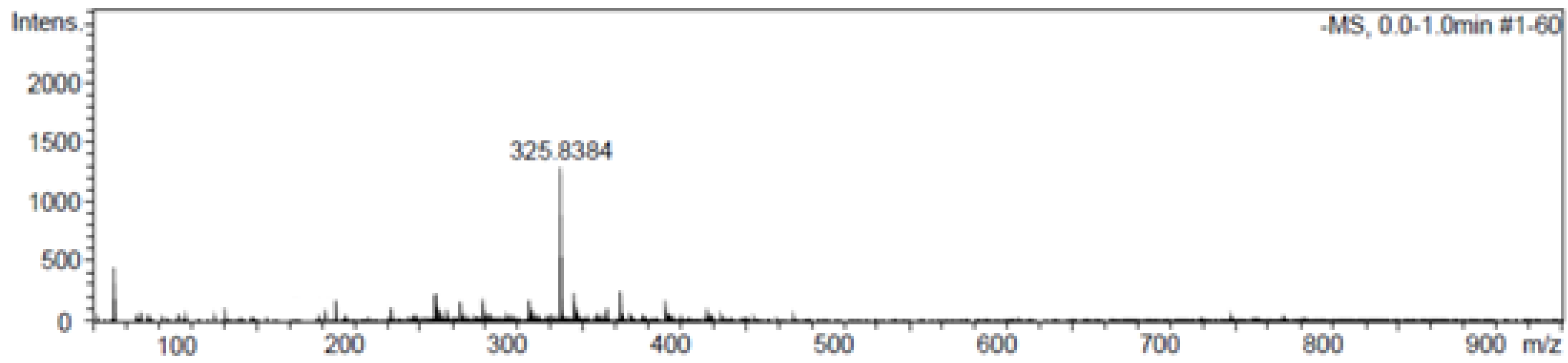

Figure S2. HRESIMS spectrum (negative mode) of compound **1**.

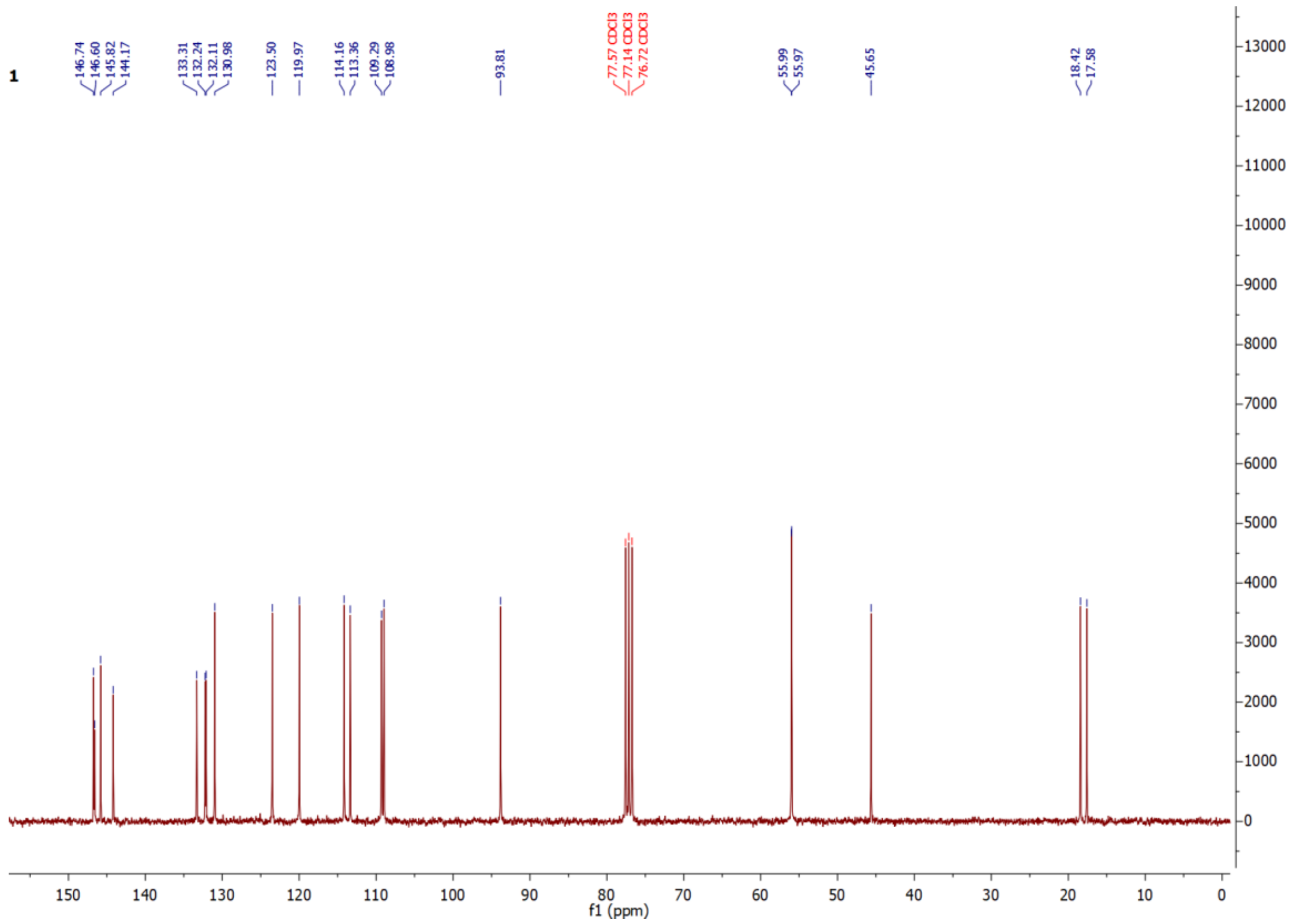

Figure S3. ^{13}C NMR spectrum of compound **1** (δ/ppm, 75 MHz, $CDCl_3$).

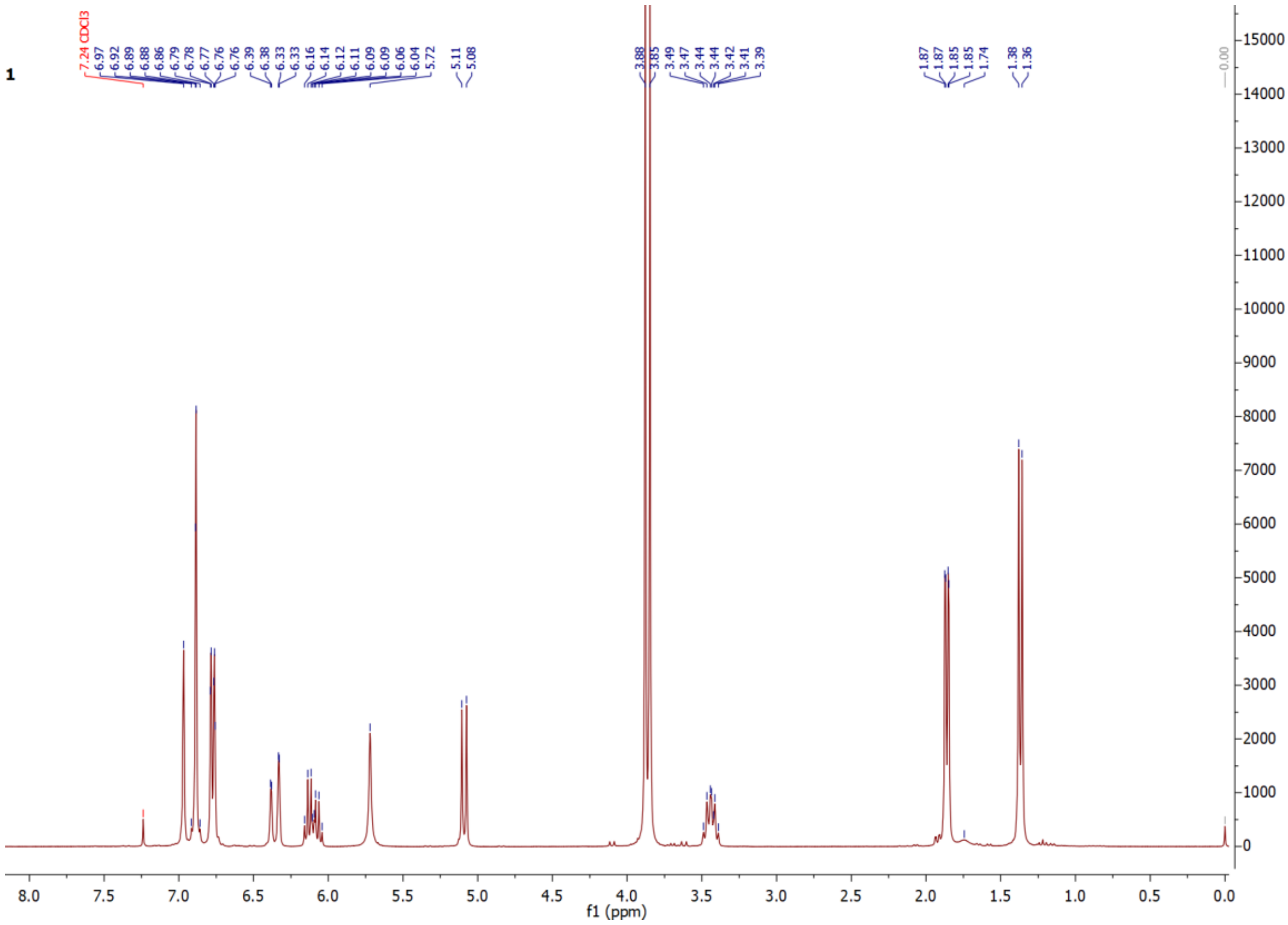

Figure S4. ¹H NMR spectrum of compound **1** (δ/ppm, 300 MHz, $CDCl_3$).

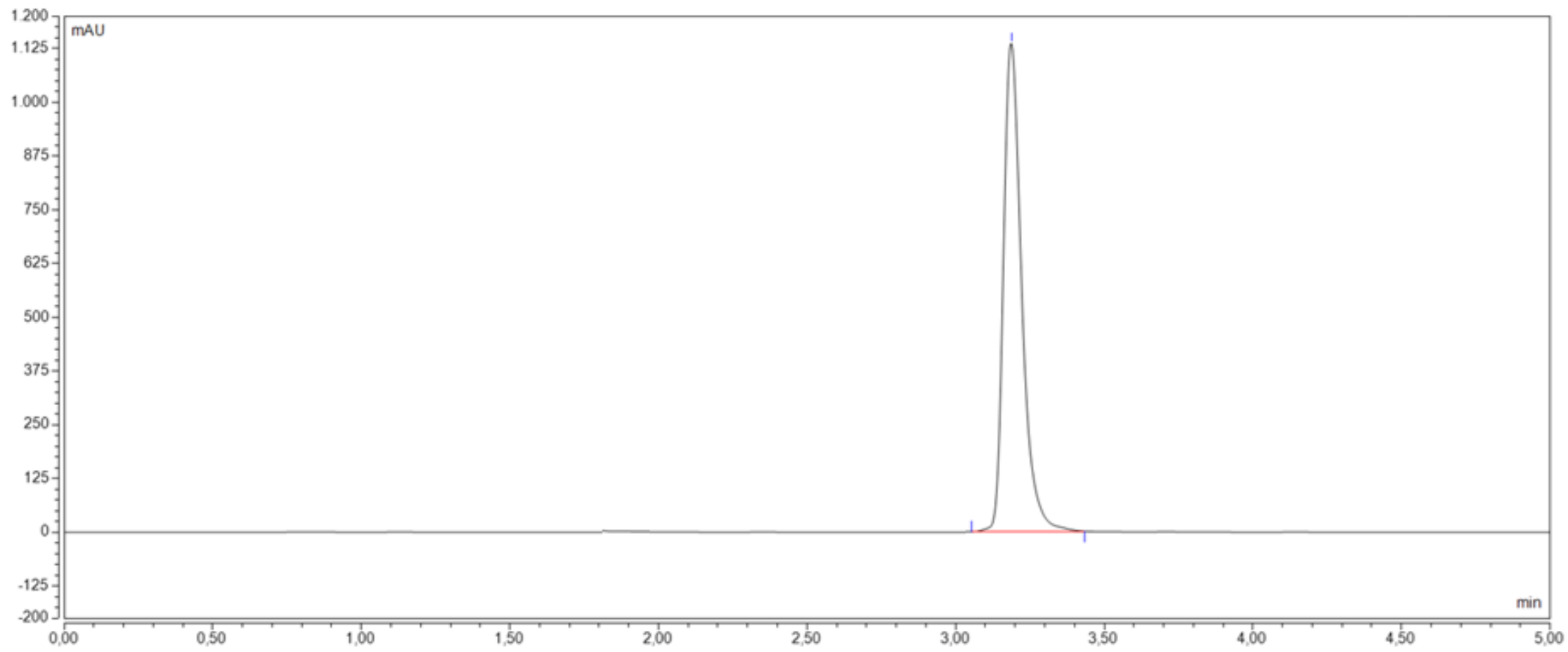

Figure S5. HPLC analysis of compound **1a**.

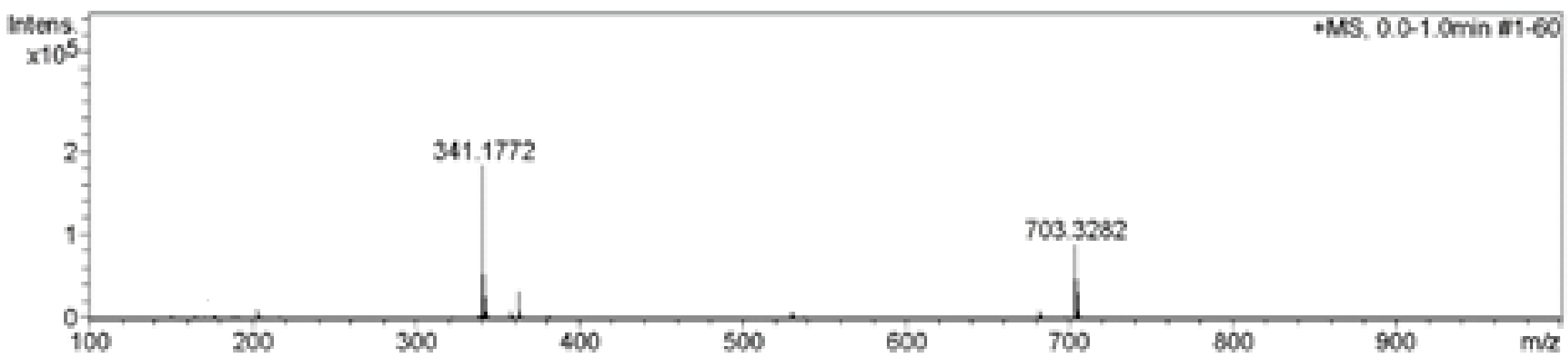

Figure S6. HRESIMS spectrum (positive mode) of compound **1a**.

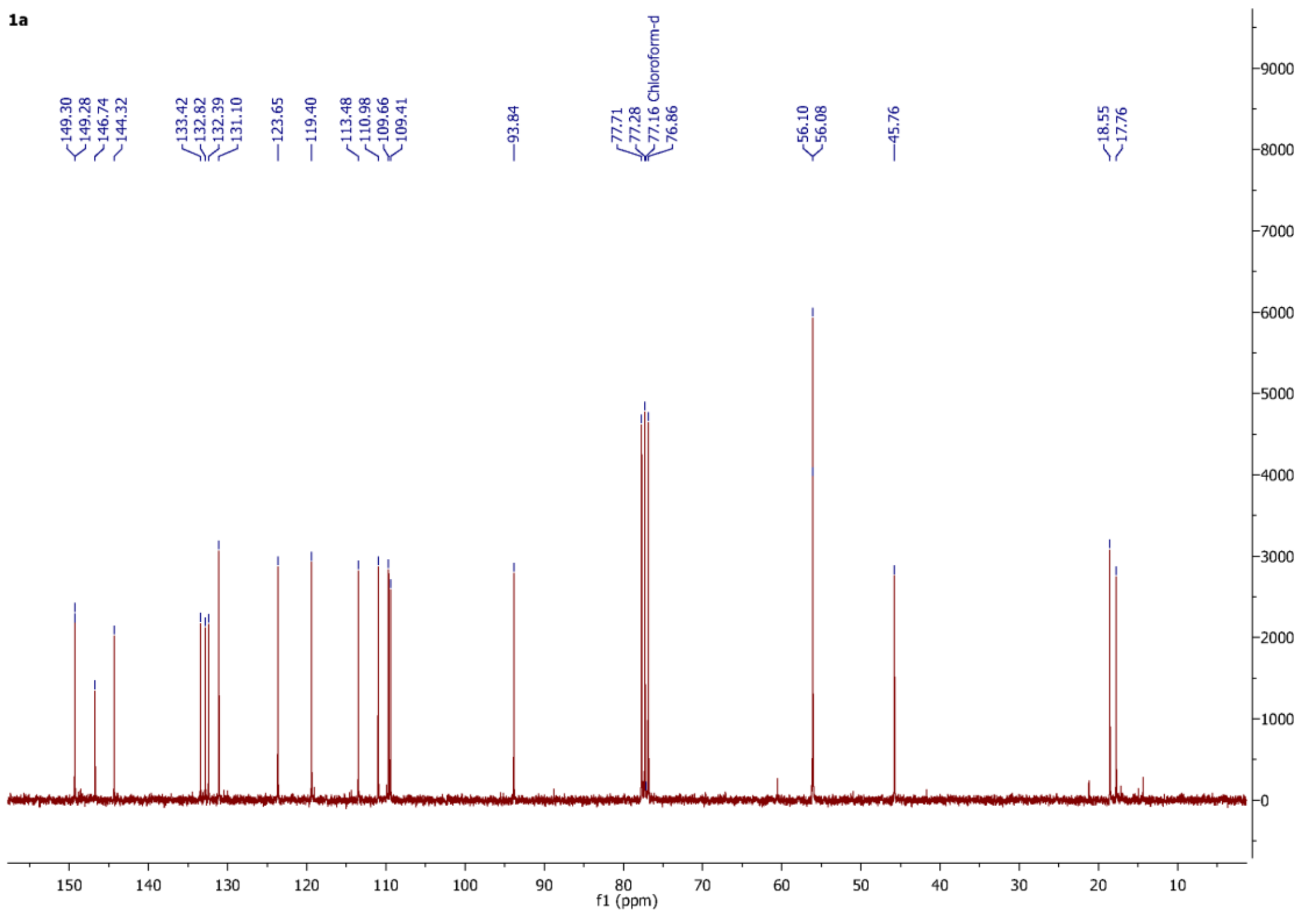

Figure S7. ^{13}C NMR spectrum of compound **1a** (δ/ppm, 75 MHz, $CDCl_3$).

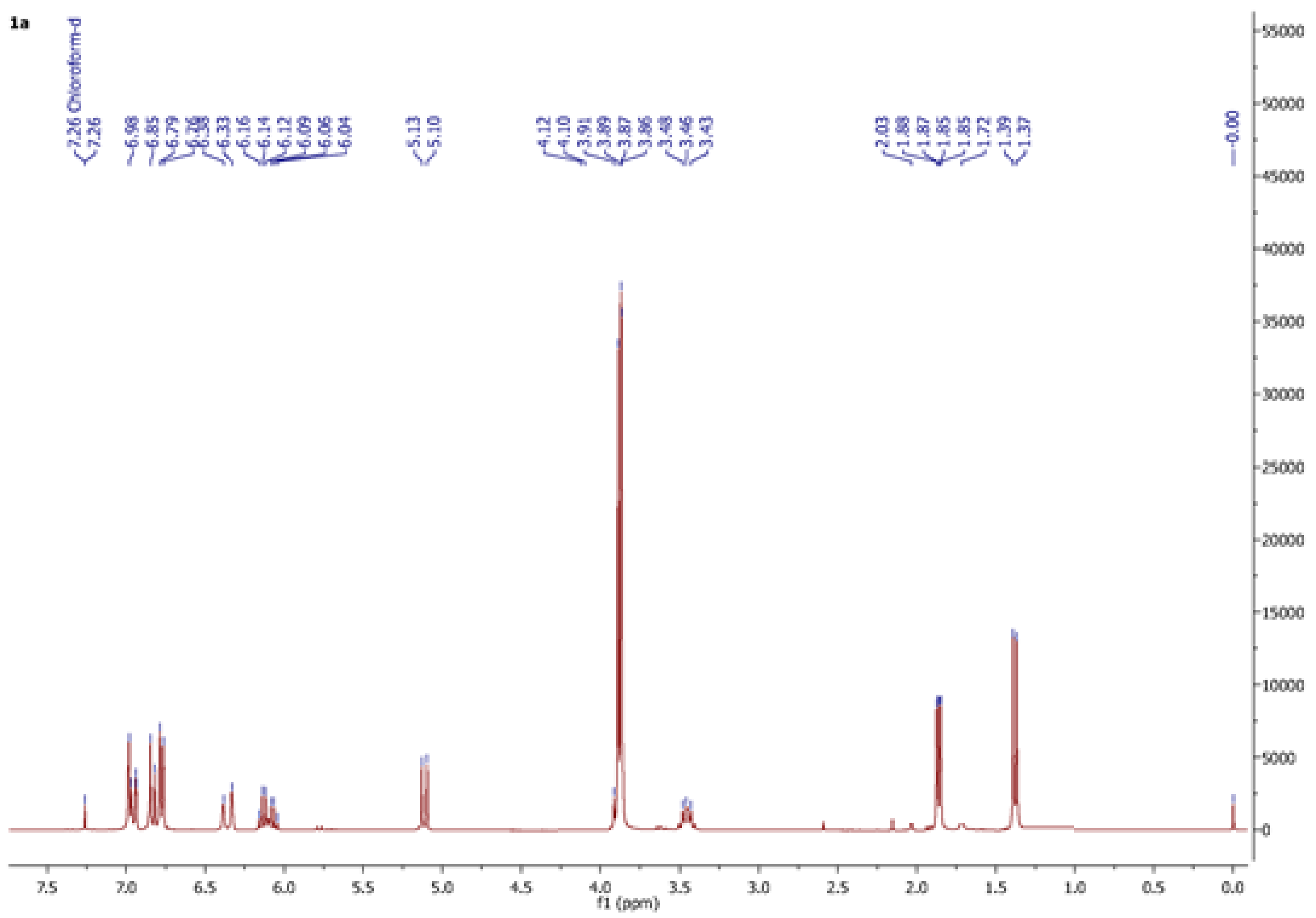

Figure S8. ^{1}H NMR spectrum of compound **1a** (δ/ppm, 300 MHz, $CDCl_3$).

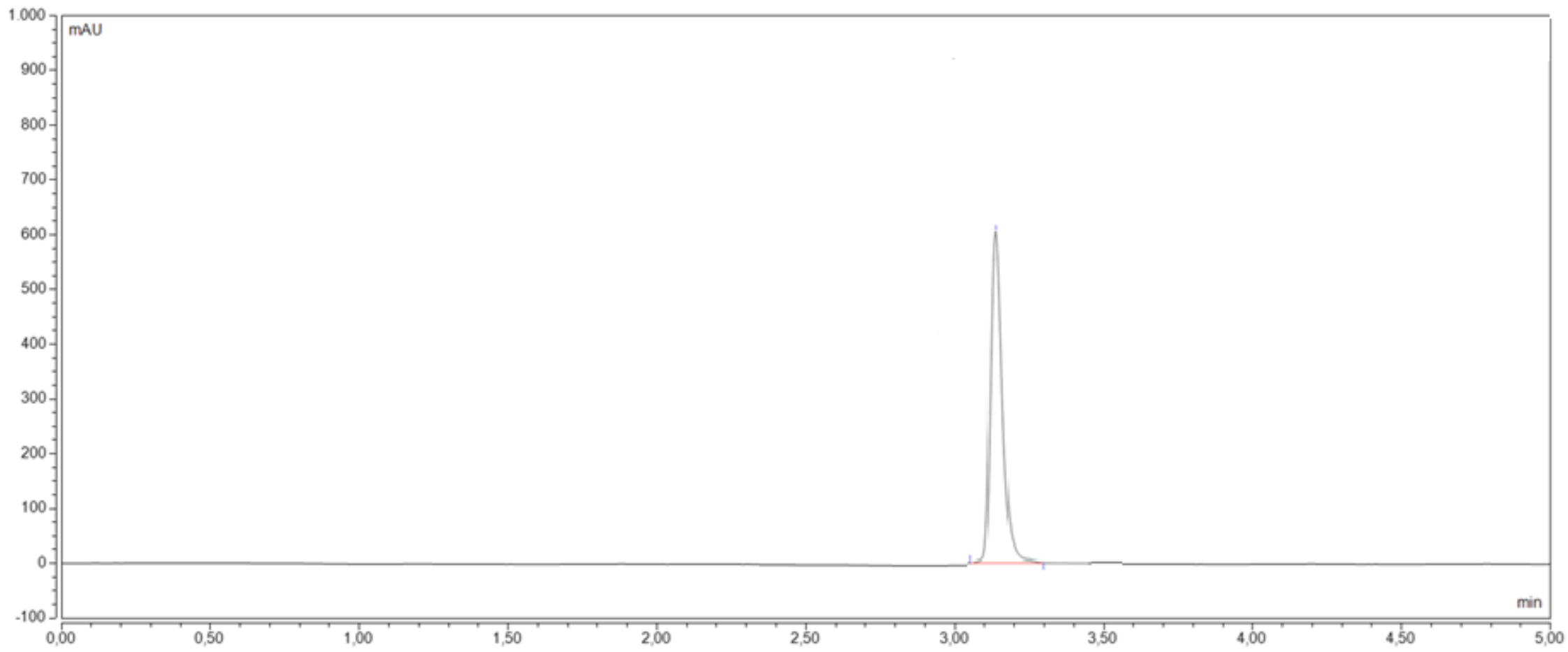

Figure S9. HPLC analysis of compound **1b**.

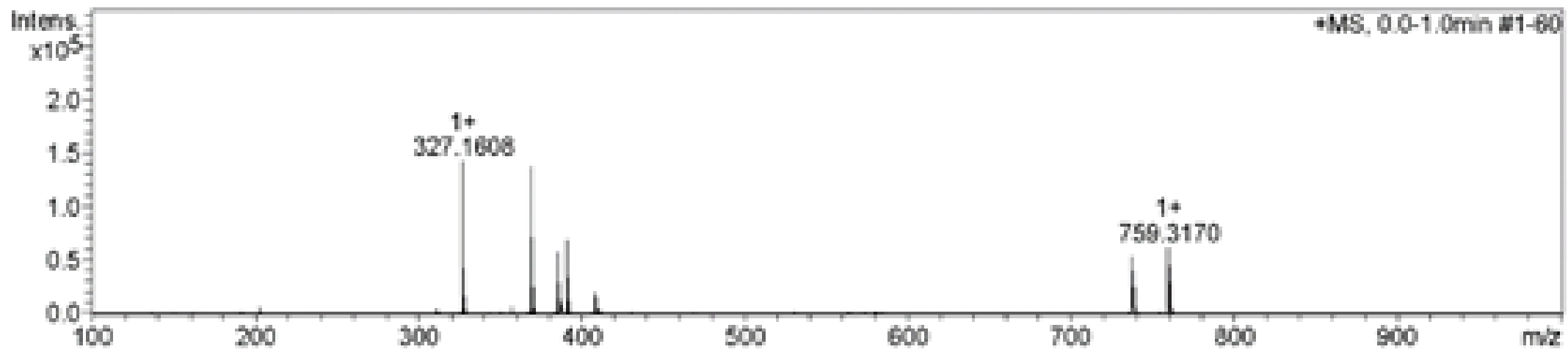

Figure S10. HRESIMS spectrum (positive mode) of compound **1b**.

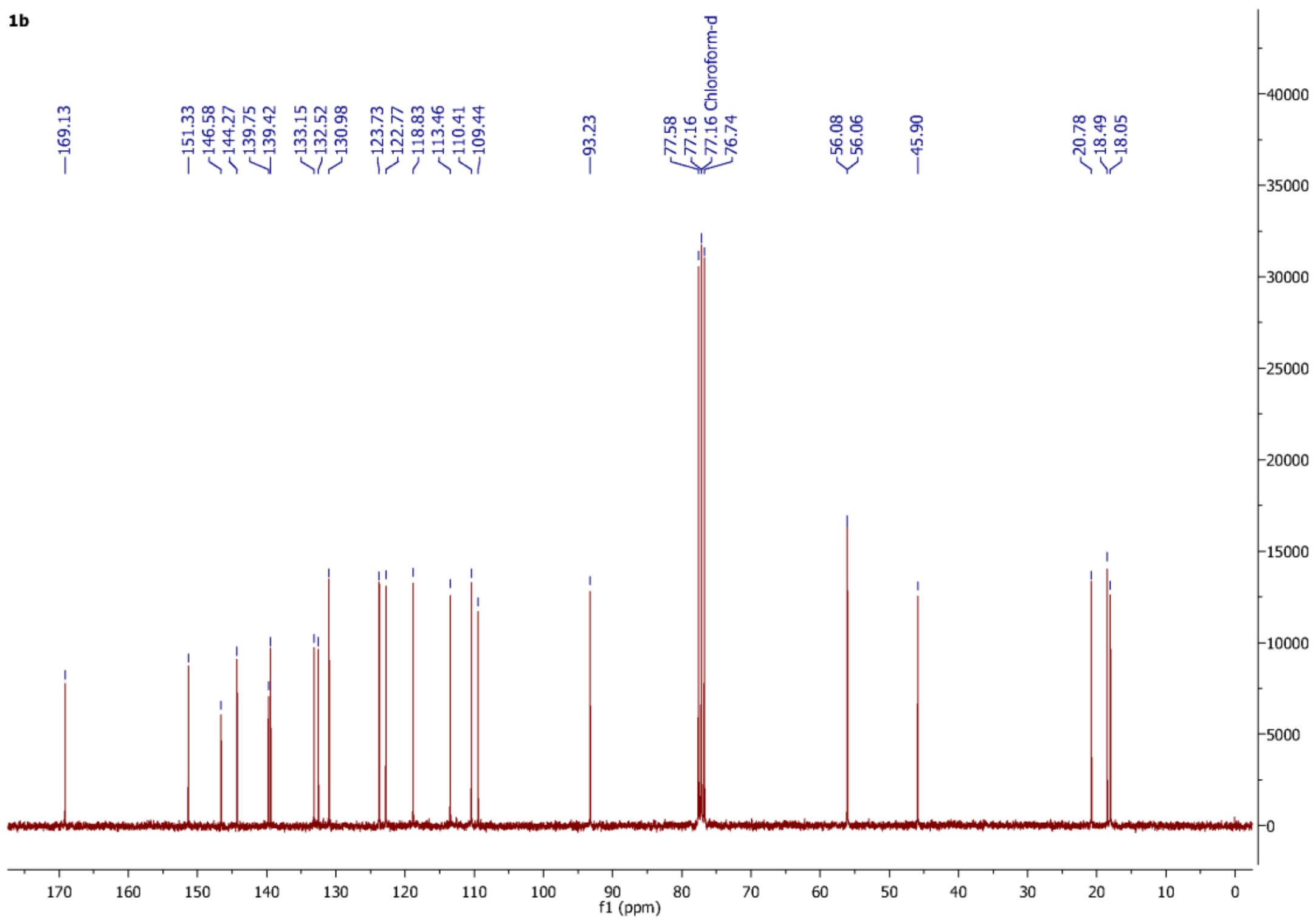

Figure S11. ^{13}C NMR spectrum of compound **1b** (δ/ppm, 75 MHz, $CDCl_3$).

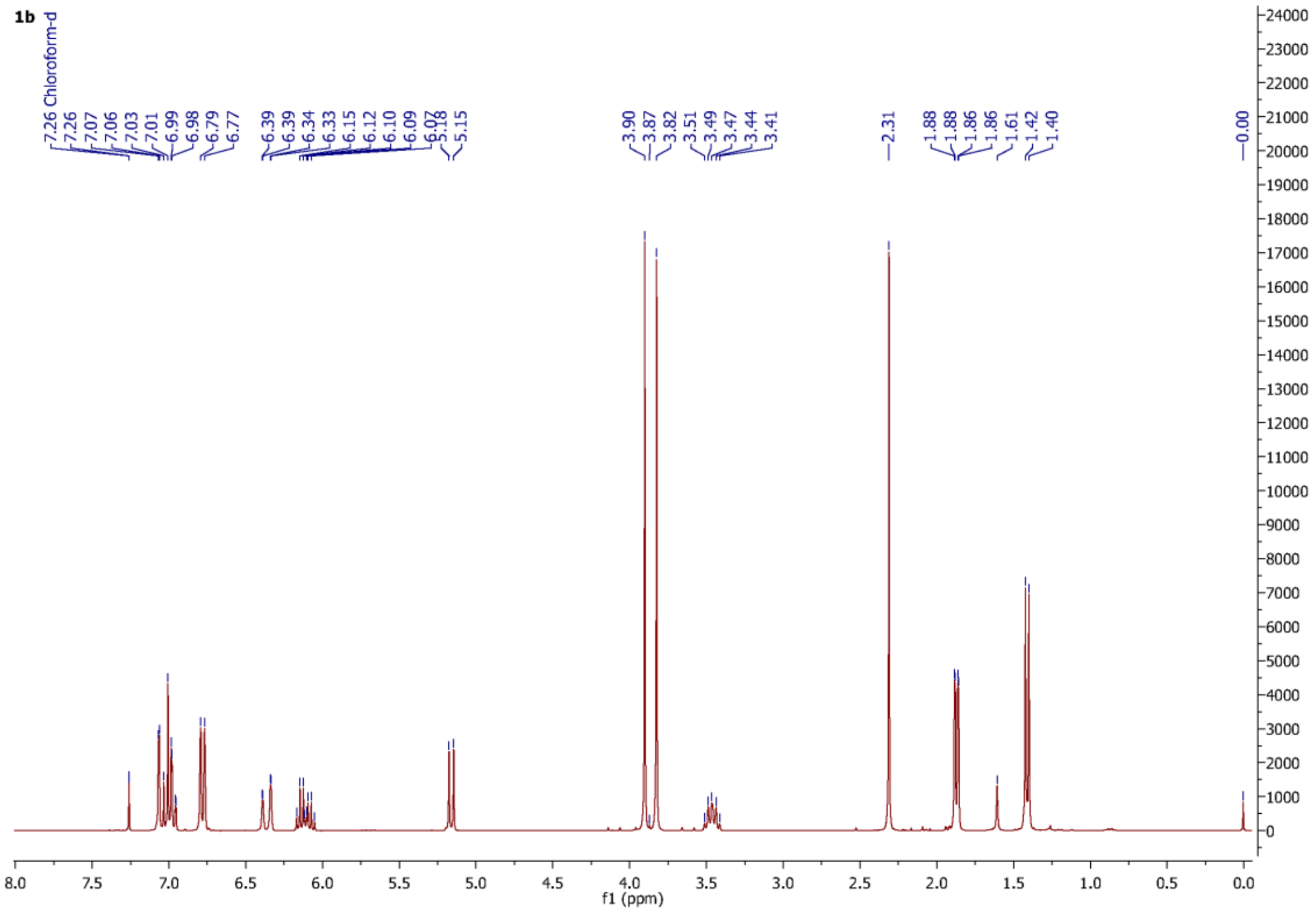

Figure S12. ^{1}H NMR spectrum of compound **1b** (δ/ppm, 300 MHz, $CDCl_3$).

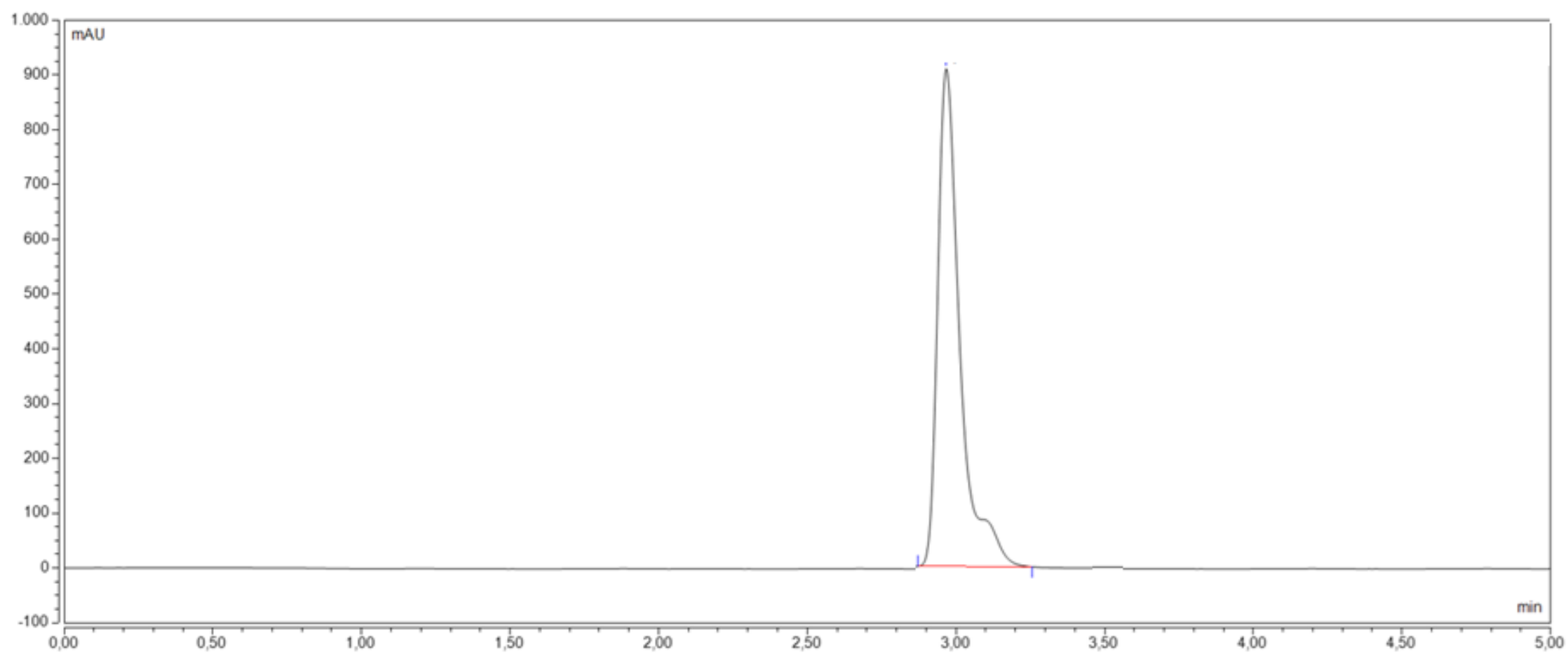

Figure S13. HPLC analysis of compound **1c**.

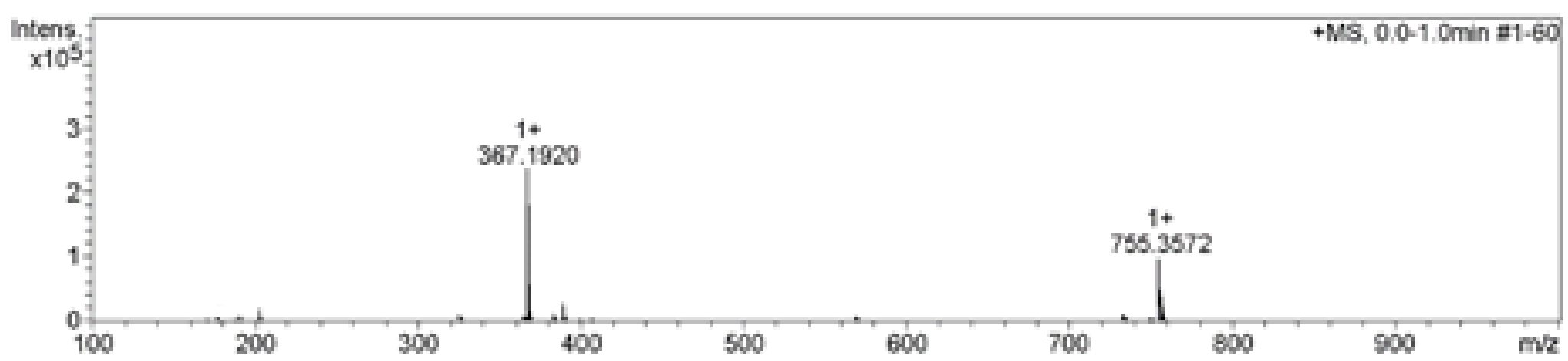

Figure S14. HRESIMS spectrum (positive mode) of compound **1c**.

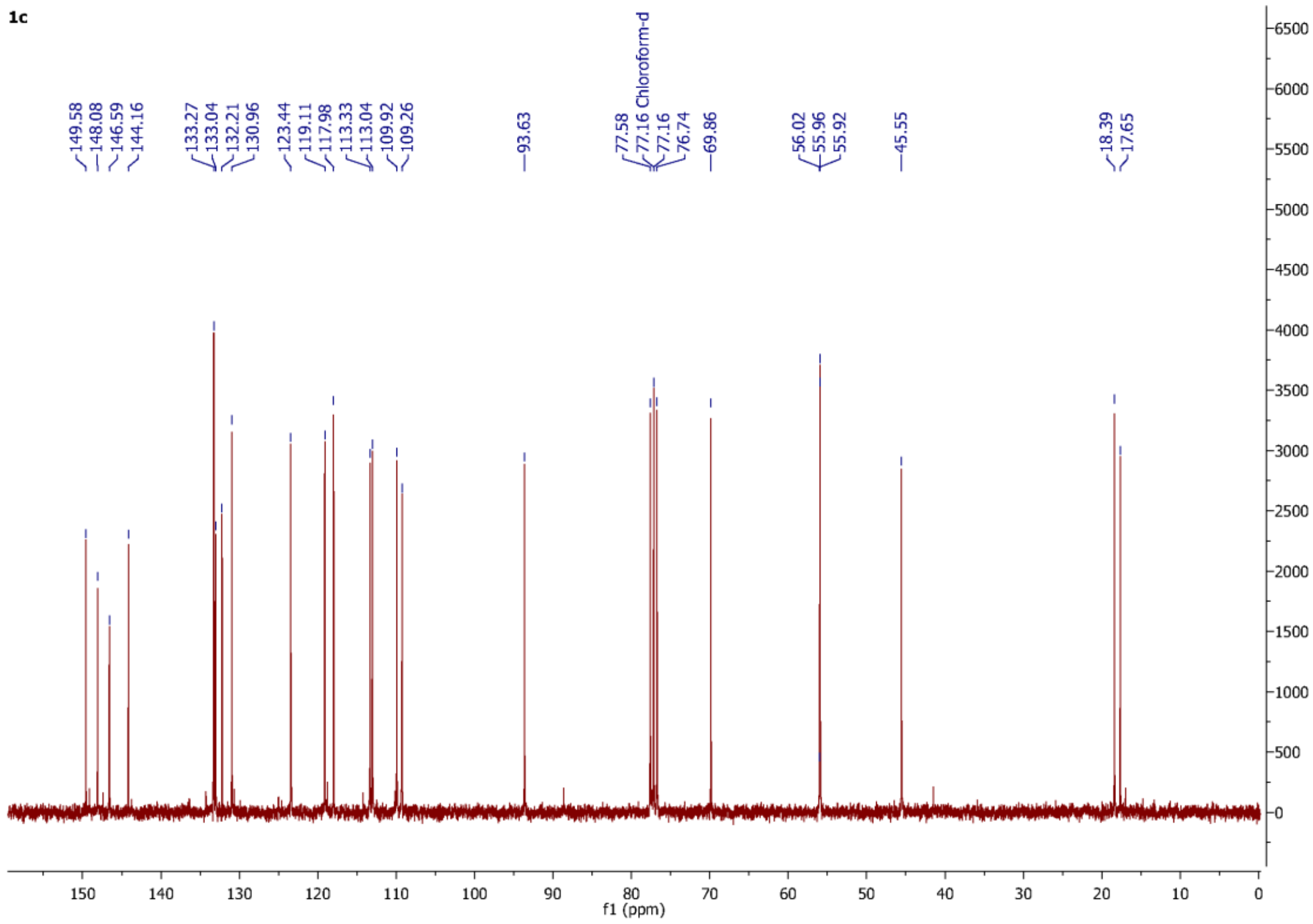

Figure S15. ^{13}C NMR spectrum of compound **1c** (δ/ppm, 75 MHz, $CDCl_3$).

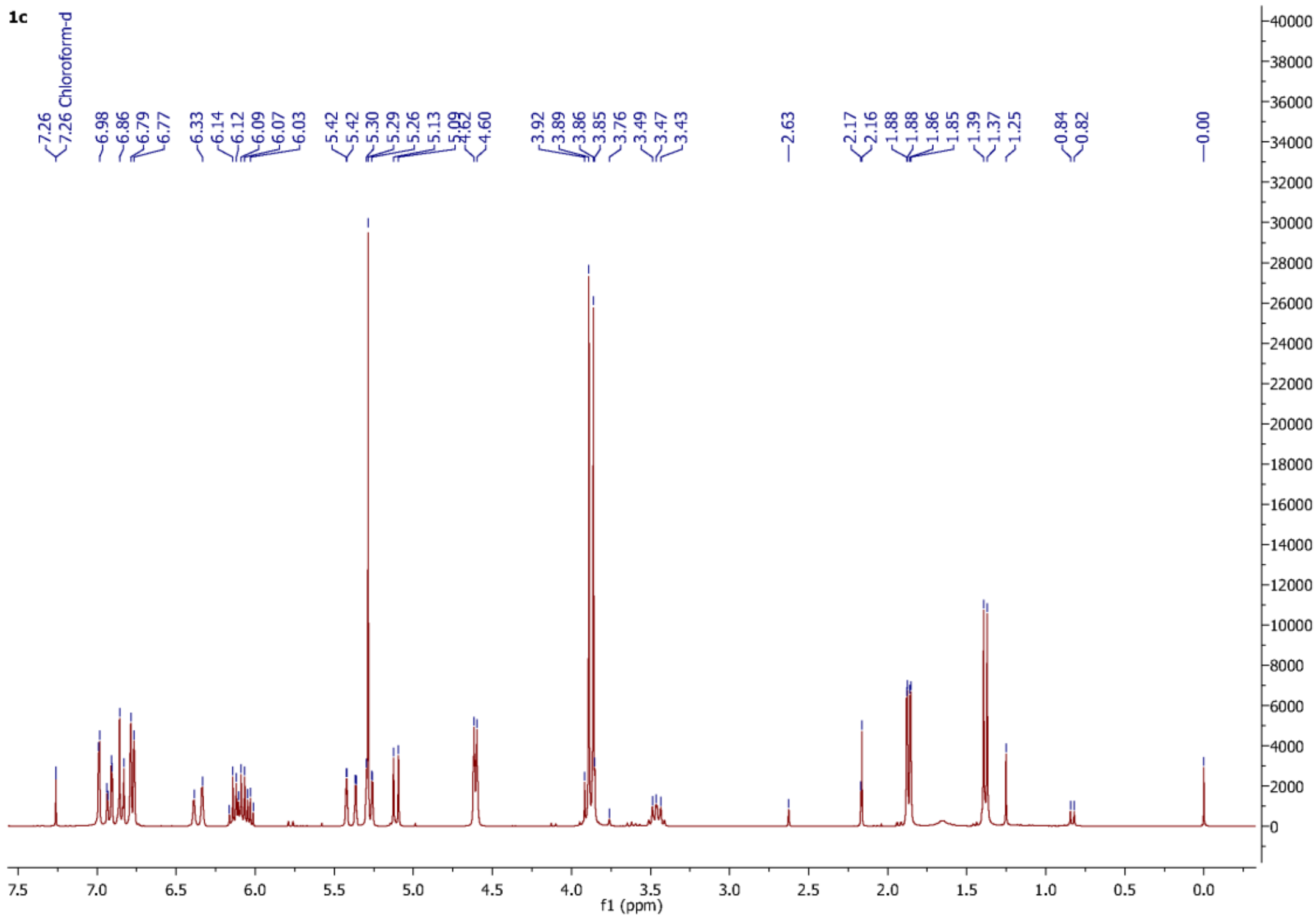

Figure S16. ^{1}H NMR spectrum of compound **1c** (δ/ppm, 300 MHz, $CDCl_3$).
